# Comparison of primordial germ cell differences at different developmental time points in chickens

**DOI:** 10.5713/ab.24.0283

**Published:** 2024-08-05

**Authors:** Wei Gong, Yichen Zou, Xin Liu, Yingjie Niu, Kai Jin, Bichun Li, Qisheng Zuo

**Affiliations:** 1 Joint International Research Laboratory of Agriculture and Agri-Product Safety of Ministry of Education of China, Yangzhou University, Yangzhou, Jiangsu 225009, China; 2 Key Laboratory of Animal Breeding Reproduction and Molecular Design for Jiangsu Province, College of Animal Science and Technology, Yangzhou University, Yangzhou, Jiangsu 225009, China

**Keywords:** Cell Proliferation, Chicken, Primordial Germ Cell, Resource Conservation, RNA-seq

## Abstract

**Objective:**

Recently, the application in the field of germplasm resource conservation has become an important application of primordial germ cells (PGCs). However, due to the lack of deep understanding of the biological characteristics of PGCs at different time points, there is no systematic scheme for the selection of PGCs at which time points in practical application, which affects the practical application effect of PGCs. This study aims to clarify the differences in PGCs during development.

**Methods:**

Here, migration experiment, EdU proliferation assay and cell apoptosis assay were conducted to compare the differences in the migration ability, the proliferation ability and the recovery efficiency among female and male PGCs at E3.5, E4.5, and E5.5, which were explained by the following transcriptome sequencing analysis.

**Results:**

We found that there were larger differences between female and male PGCs at different embryonic ages, while smaller differences between female and male PGCs at the same embryonic age. Further comparison showed that the cell migration ability of female and male PGCs decreased gradually during development, so female and male PGCs at E3.5 are more suitable for *in vitro* allotransplantation. At the same time, the proliferation ability of PGCs gradually decreased during development, and cell adhesion and extracellular matrix communication were weakened, indicating that female and male PGCs of E3.5 are more suitable for in vitro long-term culture cell line establishment. Interestingly, female and male PGCs at E5.5 showed strong DNA damage repair ability, thus more suitable for *in vitro* long-term cryopreservation.

**Conclusion:**

This study provides a theoretical basis for systematically selecting PGCs at suitable developmental time points as cell materials for efficient utilization by analyzing the characteristics of female and male PGCs at different developmental time points based on transcriptome.

## INTRODUCTION

The situation of species extinction has become serious because of the increasing exploitation of natural resources by human society and the destruction of ecological resources. In recent years, more and more frontline researchers have begun to focus on the effective conservation and restoration of species resources. Due to the gradual breakthrough of technologies including somatic cell nuclear transplantation, cryopreservation and thawing of cells and tissues, the conservation situation of mammalian germplasm resources is relatively optimistic. Species differences exist between birds and mammals, so the technology of somatic cell nuclear transplantation is difficult to apply in birds. Although the genetic material of roosters can be conserved through freezing their semen, the entire chicken genome cannot be conserved due to loss of the W chromosome. The problem of freezing oocytes or early embryos has not been solved and simultaneously the cryopreservation of gonads also faces the challenge of tissue transplantation and producing purebred offspring. Primordial germ cells (PGCs) with the ability to migrate directionally have aroused the interest of scientific researchers, and related studies found that PGCs isolated *in vitro* from Gifujidori fowl (black-feathered) were able to migrate to the genital ridges, leading to the formation of reproductive chimeras after reinjected into the blood circulation system of the donor White Leghorn chickens (white-feathered) and after sexual maturity, purebred black chickens could be generated through mating [1], which indicated that PGCs can be well applied in animal germplasm conservation.

In recent years, more and more studies have focused on the application of PGCs in germplasm conservation. Saito et al [2] achieved allotransplantation and production of normal gametes after injecting PGCs from different species of fish into zebrafish embryos. Chuma et al [3] found that mouse PGCs transplanted into the testes of sterile mice could develop into normal sperms which were able to fuse with the egg to obtain fertile offspring. Asaoka et al [4] successfully obtained fertile offspring after transplanting frozen *Drosophila* PGCs into embryos. Interestingly, in these studies we find a series of questions. Naito et al [5] injected PGCs into recipient embryos to produce chimeric chickens and found that donor PGCs injected into opposite-sex recipient embryos produced few donor offspring but when donor and recipient embryos were of the same sex, the generation efficiency of donor offspring was significantly improved, which means that the ability of PGCs to differentiate into functional gametes was impaired after transplantation into opposite-sex recipient embryos. Nakamura et al [6] found that after 60 h of incubation, the number of PGCs in the embryonic gonad region was significantly higher in female embryos than in male embryos. During *in vitro* culture of PGCs, researchers found that male PGCs proliferated faster than female PGCs and that female PGCs tended to cluster [7]. Therefore, in view of the differences in self-renewal ability of PGCs *in vitro*, the culture system was optimized for different genders [8]. It is evident that female and male PGCs differ in terms of multiple abilities at different embryonic ages. Therefore, understanding the characteristics of female and male PGCs at different developmental time points can guide us to utilize PGCs more efficiently.

RNA-seq provides us with strong evidence or new ideas to explore the regulatory mechanisms of various animal biological processes by detecting changes on the gene transcription level, and it has been widely used in recent years because of its high accuracy and efficiency. In the study of Zhao et al [9], by comparing transcriptome data of mouse and human oocytes, the limitations of using mice as a model to study human oocyte maturation were revealed. Ge et al [10] performed RNA-seq of the liver after inducing diabetes in mice with streptozotocin and successfully rationalized the pathogenesis of diabetes. Ding et al [11] found that autophagy is activated during the formation of chicken PGCs by RNA-seq and subsequent validation experiments. In another study, based on the transcriptomic analysis, small molecule compounds were added to activate Wnt and cAMP pathways or inhibit MAPK pathway during the differentiation of embryonic stem cells to PGC-like cells, and the induction system of chicken PGCs *in vitro* was successfully optimized [12].

To select female or male PGCs at appropriate developmental time points for utilization, female and male PGCs at E3.5, E4.5 and E5.5 were collected respectively the characteristics of which were compared based on transcriptome sequencing, and it was found that female and male PGCs at different embryonic ages were different in terms of proliferation, migration, etc. These results provide a theoretical basis for the practical application of PGCs.

## MATERIALS AND METHODS

### Ethics statement

All of the procedures involving the care and use of animals were approved by the Institutional Animal Care and Use Committee of Yangzhou University (approval number: SYXK [Su] 2021–0027). The fertilized eggs of Rugao yellow chicken were purchased from the Poultry Research Institute of Chinese Academy of Agricultural Sciences and hatched under the environment of 37°C with 70% humidity.

### Cell isolation and culture

The freshly fertilized eggs from Rugao yellow chickens were provided by the Poultry Research Institute of CAAS, Yangzhou, Jiangsu Province, China. PGCs were isolated from the genital ridges of chicken embryos incubated for 3.5 days, 4.5 days, 5.5 days respectively and cultured in Dulbecco’s modified eagle medium (21068028; Gibco, Carlsbad, CA, USA) containing 24% H_2_O (W3500; Sigma-Aldrich, St. Louis, MO, USA), 0.15 mM CaCl_2_ (C7902; Sigma-Aldrich, USA), 1% GlutaMax (35050061; Gibco, USA), 1% non-essential amino acids (11140050; Gibco, USA), 1.2 mM sodium pyruvate (11360070; Gibco, USA), 0.1 mM β-mercaptoethanol (21985023; Gibco, USA), 0.2% chicken serum (16110082; Gibco, USA), 0.2% ovalbumin (A5503; Sigma-Aldrich, USA), 2% B-27 (17504044; Gibco, USA), 1% EmbryoMax Nucleosides (ES-008; Sigma-Aldrich, USA), 0.01% heparin sodium (HY-17567A; MCE, Monmouth Junction, NJ, USA), 25 ng/mL activin A (HY-P70311; MCE, USA), 4 ng/mL FGF-2 (HY-P70600; MCE, USA), 1% penicillin–streptomycin (15140148; Gibco, USA). PGCs were maintained in a 5% CO_2_ humidified atmosphere at 37.0°C. The isolation and culture methods for chicken PGCs have been described previously [13].

### Sex identification

Chicken embryo blood was added to the reaction solution of Mighty Amp DNA Polymerase (R071A; Takara, Dalian, China). Amplification was performed using *CHD1* specific primers (F: CTGCGAGAACGTGGCAACAGAGT; R: ATTGAAATGATCCAGTGCTTG) to identify the sex of the embryos and agarose gel electrophoresis showed two strips at 580 bp and 434 bp for female (ZW) and only one strip at 580 bp for male (ZZ) [14].

### Flow cytometry

Female and male PGCs at E3.5, E4.5, E5.5 were collected and washed with phosphate buffered saline (PBS). Then 1% Triton X-100 was added for 15 min to permeabilize the membrane, and the cells were blocked with 10% fetal bovine serum (FBS) for 2 h. C-KIT antibody was added and incubated at 37°C for 2 h and then at 4°C overnight. The secondary antibody was added after washing the cells three times with PBS-Tween (PBST) and incubated at 37°C for 2 h without light. The cells were resuspended with PBS and detected using flow cytometry (BD LSRFortessa; BD Biosciences, San Jose, CA, USA).

### Periodic acid-Schiff (PAS) staining

The collected PGCs were resuspended with PAS fixative and dropped onto cell slides to dry in a ventilated area. The cells were washed with distilled water and air-dried, and stained with PAS staining kit (G1360; Solarbio, Beijing, China). The cells were treated with oxidant for 10 min at room temperature and then washed with distilled water and air-dried. Then 20 μL Schiff reagent per slice was used to stain the cells for 15 min away from light, and the cells were washed with sodium sulfite solution and distilled water. After air-dried, the cells were stained with hematoxylin for 2 min.

### Indirect immunofluorescence

The collected female and male PGCs at E3.5, E4.5, E5.5 were fixed with 4% paraformaldehyde for 30 min, washed with PBS three times and treated with 1% Triton X-100 for 15 min, followed by the addition of 10% FBS to block the cells for 2 h. Then, the primary antibodies against chicken vasa homologue (CVH) (ab13840; Abcam, Cambridge, MA, USA) and SSEA-1 (ab16285; Abcam, USA) were added and incubated at 37°C for 2 h and then at 4°C overnight. After washing three times with PBST, the cells were incubated with the corresponding secondary antibodies at 37°C for 2 h without light, then washed three times with PBST, followed by incubation with 4’,6-diamidino-2-phenylindole (DAPI) for 10 min without light and then washed. Fluorescence was observed and photographed under the fluorescence microscope (FV1200; Olympus, Tokyo, Japan).

### Quantitative real-time polymerase chain reaction

Total RNA was extracted from 2×10^6^ female and male PGCs at E3.5, E4.5, E5.5 and chicken embryonic fibroblasts (CEFs) respectively using TRNzol (DP424; Tiangen, Beijing, China) and reverse transcribed into cDNA with HiScript III RT SuperMix (R323–01; Vazyme, Nanjing, China). *ACTB* was used as an internal control gene to estimate the expression of *CVH* and *POU5F3* [15–17]. The reaction mixture for quantitative real-time polymerase chain reaction (qRT-PCR) included 10 μL 2×ChamQ Universal SYBR qPCR Master Mix, 0.6 μL each of upstream and downstream primer, 2 μL cDNA and 6.8 μL ddH_2_O. The reaction procedure was performed according to the instructions provided by ChamQ Universal SYBR qPCR Master Mix (Q711–02; Vazyme, China). The relative gene expression was calculated with the 2^−ΔΔCt^ method [18]. The primers are supplied in Supplementary Table S10.

### RNA-seq data analysis

Total RNA was extracted from 5×10^7^ female and male PGCs at E3.5, E4.5, E5.5 using TRNzol (DP424; Tiangen, China). RNA purity and concentration were evaluated with the NanoDrop 2000 spectrophotometer (Thermo Scientific, Waltham, MA, USA). RNA integrity was assessed using the Agilent 2100 Bioanalyzer (Agilent Technologies, Santa Clara, CA, USA). The libraries were constructed with VAHTS Universal V6 RNA-seq Library Prep Kit and sequenced on an Illumina Novaseq 6000 platform by Shanghai OE Biotechnology (Shanghai, China). The raw data were quality assessed and filtered by FastQC to obtain high quality and relatively accurate valid data [19]. Gene expression (fragments per kilobase of exon model per million mapped fragments [FPKM] value) was analyzed by Cuffdiff after comparing the valid data of the samples with the reference genome using HISAT2 [20], and differentially expressed genes were screened by p<0.05 and |log2FC| >1. The process and method of gene ontology (GO) [21] and Kyoto encyclopedia of genes and genomes (KEGG) [22] enrichment analysis for differentially expressed genes were referred to relevant studies.

### Migration experiment

The green fluorescent protein (GFP)^+^ PGCs at E3.5, E4.5, and E5.5 were obtained according to the previous study [23]. When the recipient chicken embryos developed to E2.5 (HH17), a small hole was made at the blunt end of the egg and a window, approximately 1 cm in diameter, was created with forceps. A total of 5,000 GFP^+^ PGCs were injected into the dorsal aorta of the recipient embryo under a stereomicroscope and 20 μL penicillin–streptomycin was dropped into the window. Then the window was sealed with medical breathable tape, and the eggs were put back to the incubator for incubation. The recipient chicken embryos were collected after developing to E7.5 (HH32) and the migration of GFP^+^ PGCs to the gonads was observed under a stereoscopic fluorescence microscope.

### EdU proliferation assay

Female and male PGCs at E3.5, E4.5, E5.5 were inoculated in 24-well plates with 1×10^5^ per well and labeled with 50 μL of 50 μM EdU medium. Then the cells were fixed with 4% paraformaldehyde, followed by Apollo (C10310–1; RiboBio, Guangzhou, China) staining. Finally, DNA was stained with Hoechst33342 (C10310–1; RiboBio, China) and observed under the fluorescence microscope.

### Cell apoptosis assay

After 5×10^5^ female and male PGCs at E3.5, E4.5, E5.5 were collected and washed with pre-cooled PBS, the cells were respectively incubated with Annexin V-fluorescein isothiocyanate (FITC) and propidium iodide (PI) (40302ES50, Yeasen, Shanghai, China) at room temperature for 15 min without light. The cells were detected by flow cytometry.

### Data analysis

All experiments were repeated at least three times, and the data are presented as mean±standard error. After all data sorted by EXCEL, SPSS 19.0 (SPSS, Chicago, IL, USA) and Graph Pad Prism 6 (GraphPad Software Inc., San Diego, CA, USA) were used to perform significance analysis and generate diagrams. Significant differences between the groups were determined by one-sample *t*-test and one-way analysis of variance (* p<0.05, significant difference. ** p<0.01, extremely significant difference).

## RESULTS

### Isolation, culture and identification of male and female PGCs at E3.5, E4.5, and E5.5

**Figure 1:**
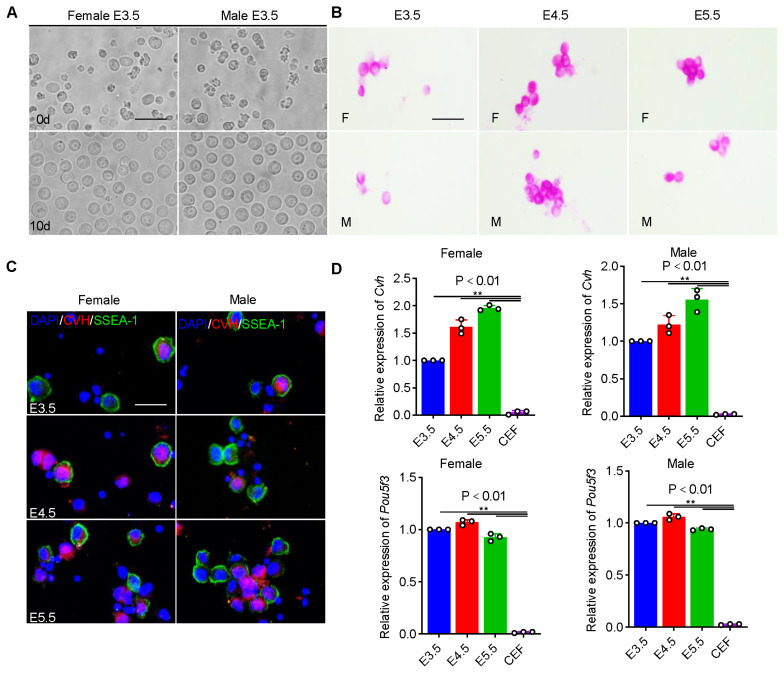
Isolation, culture and identification of male and female PGCs at E3.5, E4.5, and E5.5. (A) Morphological observation of female and male PGCs at E3.5 after isolation and purification. Scale bar: 60 μm. (B) PAS staining to identify female and male PGCs at E3.5–E5.5. Scale bar: 60 μm. (C) Female and male PGCs at E3.5–E5.5 were identified by indirect immunofluorescence detection with CVH and SSEA-1 antibody. Scale bar: 60 μm. (D) qRT-PCR was used to detect the expression of *CVH* and *POU5F3* in female and male PGCs at E3.5–E5.5 (Data are shown as mean±standard error of the mean, n = 3 independent experiments, ** p<0.01, * p<0.05, one-way analysis of variance). CEFs were used as control. PGCs, primordial germ cells; PAS, Periodic acid-Schiff; qRT-PCR, quantitative real-time polymerase chain reaction.

To effectively isolate PGCs *in vivo*, we first analyzed the proportion of PGCs in female and male genital ridges at E3.5, 4.5, and 5.5 by flow cytometry. The results showed that the proportion of PGCs in female and male genital ridges at different developmental time points was very small but different, and there was no significant difference between the proportion of female PGCs and that of male PGCs at the same embryonic age (p>0.05). These preliminarily indicated that PGCs in the early genital ridges have a certain proliferation ability (Supplementary Figure S1B–1D). Subsequently, the female and male PGCs were cultured and purified *in vitro* using feeder layer-free culture system. Morphological observations showed that the diameter of PGCs was significantly larger than that of somatic cells; during purification culture, the number of somatic cells in the culture medium gradually decreased while the number of PGCs increased significantly, presenting as large single round clones with smooth edges (Figure 1A; Supplementary Figure S1E). The results of cell identification showed that female and male PGCs at different embryonic ages were all positive for PAS, PGC marker protein CVH and stem cell marker protein SSEA-1 (Figure 1B, 1C). The results of qRT-PCR showed that the expression of pluripotency marker gene *POU5F3* and PGC marker gene *CVH* was significantly higher in PGCs than in CEFs (p<0.01) (Figure 1D). These results indicated that high-quality female and male PGCs at different embryonic ages were successfully isolated and obtained.

### Large differences were found among PGCs of E3.5, 4.5, and 5.5, but not between males and females

To systematically identify the differences between female and male PGCs at different embryonic stages, female and male PGCs at different embryonic stages were collected and transcriptome sequencing was performed in this study. p<0.05 and |log2FC| >1 was used as the criterion for differentially expressed genes (DEGs) screening. According to the statistics of the number of DEGs, the number of DEGs between female and male PGCs was higher and increased obviously during development, but the number of DEGs between female and male PGCs at the same developmental time point was lower, showing an increasing trend (Supplementary Figure S2A). To further evaluate the differences between female and male PGCs at different developmental time points, PCA and Pearson’s correlation analysis were conducted, and the results were consistent with the statistical results of DEGs: there were larger differences between PGCs at different embryonic stages and smaller differences between male and female PGCs at the same embryonic stage (Supplementary Figure S2B, 2C).

### PGCs at early embryonic stage (E3.5) are more suitable for allotransplantation

**Figure 2:**
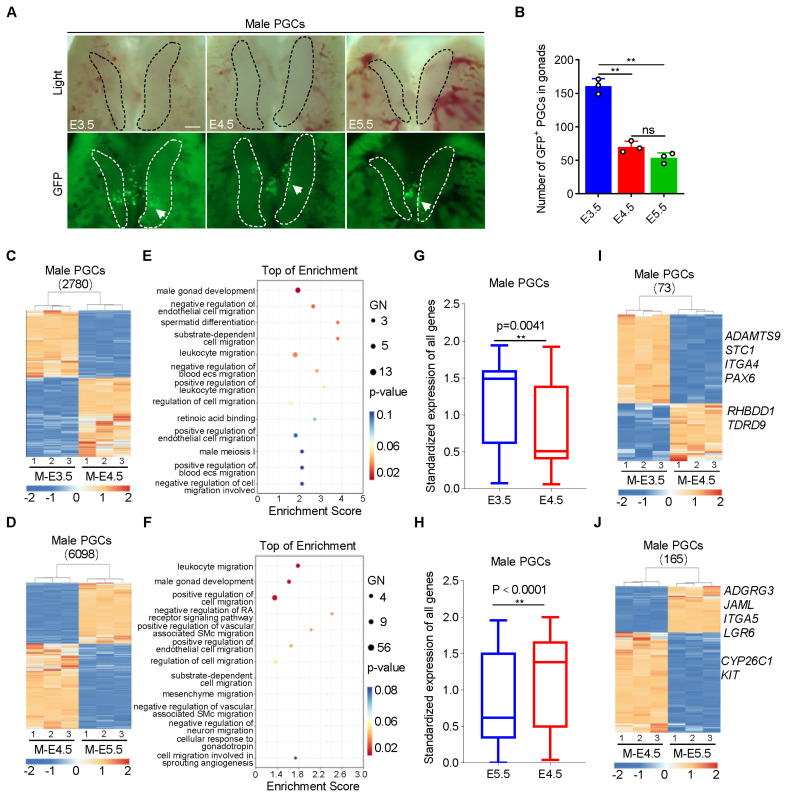
Evaluation of the migration ability of male PGCs during development. (A) Migration detection of PGCs at E3.5, E4.5, and E5.5. The dashed circles indicate the gonads, and the arrows indicate the GFP^+^ PGCs that have migrated to the gonads. Scale bar: 50 μm. (B) Statistical analysis of the number of GFP^+^ PGCs in the gonads. (C, D) Heat map analysis of DEGs during the development of male PGCs from E3.5 to E4.5 (C) and from E4.5 to E5.5 (D). (E, F) GO analysis of DEGs during the development of male PGCs from E3.5 to E4.5 (E) and from E4.5 to E5.5 (F). (G, H) Expression analysis of genes related to germline transmission ability (including genes related to migration and gametogenesis) during the development of male PGCs from E3.5 to E4.5 (G) and from E4.5 to E5.5 (H). (I, J) Heat map analysis of genes related to germline transmission ability during the development of male PGCs from E3.5 to E4.5 (I) and from E4.5 to E5.5 (J). PGCs, primordial germ cells; GFP, green fluorescent protein; GO, gene ontology; DEGs, differentially expressed genes. * p<0.05, ** p<0.01.

The allogeneic migration ability of PGCs is fundamental to the application of PGCs in transgenic and breeding fields and is also an important index to evaluate the germline transmission ability in the process of PGC allotransplantation. We compared the migration ability of PGCs at E3.5, E4.5, and E5.5 and found that the number of PGCs at E3.5 that migrated to the gonads was extremely significantly higher than that of PGCs at E4.5 or E5.5 (p<0.01) (Figure 2A, 2B), indicating that PGCs at E3.5 possess stronger migration ability. Therefore, the germline transmission ability of female and male PGCs, especially the migration capacity, was evaluated during development. The results showed that 2,780 genes were screened and identified as DEGs in male PGCs during the development from E3.5 to E4.5, of which 73 DEGs were enriched in 13 (13/62) GO terms related to germline transmission ability, such as spermatogenesis, cell migration (p< 0.1) (Figure 2C, 2E; Supplementary Table S1). Gene expression analysis revealed that the expression of genes related to germline transmission ability was higher than that in E4.5 (Figure 2G; Supplementary Table S2). Specifically, genes related to cell migration ability *ADAMTS9*, *STC1*, *ITGA4*, and *PAX6* were significantly down-regulated, probably due to the gradual loss migration ability after colonizing gonads while spermatogenesis-related genes *RHBDD1*, *TDRD9* were up-regulated, indicating that as development progressed PGCs in gonads had begun to prepare for spermatogenesis, in other words, PGCs stored materials for further germline specialization (Figure 2I). During further development (E4.5–E5.5), more DEGs (165) were significantly enriched in 13 GO terms related to germline transmission ability (Figure 2D, 2F; Supplementary Table S1). Gene expression analysis further proved that as development progressed, the germline transmission ability of PGCs declined, with migration-related genes *ADGRG3*, *JAML*, *ITGA5*, and *LGR6* down-regulated; meanwhile male germ cells were further specialized, with *KIT* up-regulated (Figure 2H, 2J; Supplementary Table S3). It is interesting to find that at this time *CYP26C1* showed a tendency to highly expressed to inhibit the progression of meiosis, which is an important mechanism to ensure the normal development of early reproduction without entering meiosis in advance. The changes in germline transmission ability during the development of female PGCs were basically consistent with those of male PGCs (Supplementary Figure S3; Supplementary Table S4–S6). Based on the results of these analyses, we conclude that early PGCs are more suitable for *in vitro* manipulation and allotransplantation.

### Female and male PGCs experience loss of proliferation capacity during development

**Figure 3:**
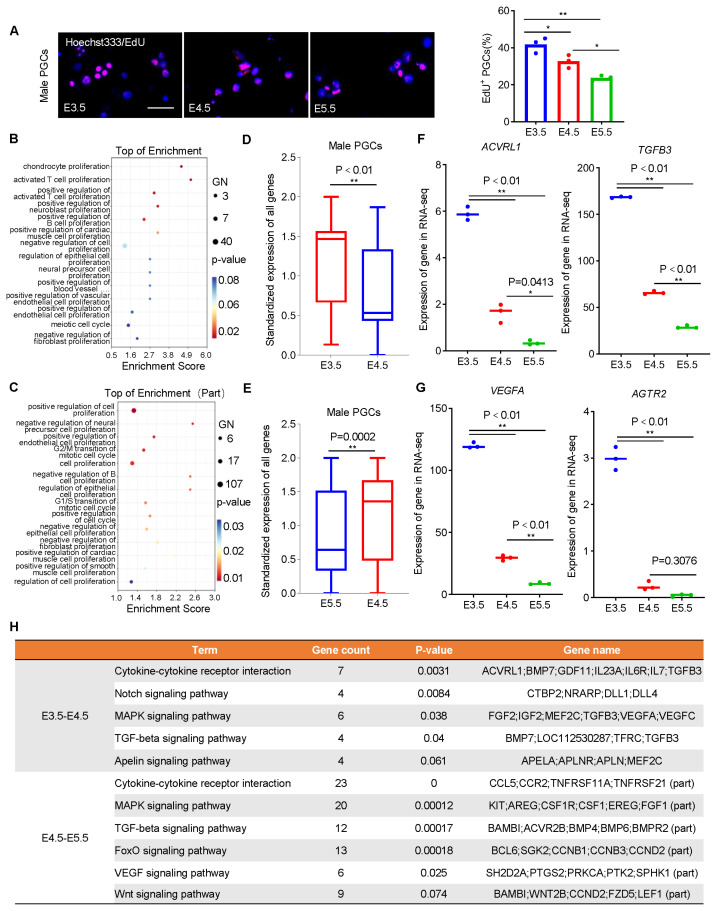
Changes in the proliferation ability of male PGCs during development. (A) EdU proliferation detection of the differences in the proliferation ability of male PGCs during *in vitro* culture. Scale bar: 60 μm. (B, C) During the development of male PGCs, 105 (E3.5–E4.5) (B) and 314 (E4.5–E5.5) (C) DEGs were significantly enriched in 14 and 32 proliferation-related GO terms respectively. (D, E) Expression analysis of genes related to proliferation during the development of male PGCs from E3.5 to E4.5 (D) and from E4.5 to E5.5 (E). (F, G) Specific expression analysis of genes related to cell proliferation during the development of male PGCs from E3.5 to E4.5 (F) and from E4.5 to E5.5 (G). (H) Signaling pathway enrichment analysis of DEGs involved in the regulation of cell proliferation during the development of chicken PGCs. PGCs, primordial germ cells; DEGs, differentially expressed genes; GO, gene ontology. * p<0.05, ** p<0.01.

It is universally acknowledged that the proliferation capacity of cells is critical for long-term culture *in vitro*. PGCs were collected for EdU proliferation assay. The results showed that the number of EdU^+^ PGCs decreased significantly with development (Figure 3A). To explain this, we compared the differences in cell proliferation capacity of male PGCs during development. Functional annotation was performed on the DEGs screened (2,780 from E3.5 to E4.5 and 6,098 from E4.5 to E5.5), which showed that during the development of PGCs from E3.5 to E5.5, respectively 105 and 314 DEGs were significantly enriched in 14 (14/74, p<0.1) and 32 (32/88, p<0.1) terms related to cell proliferation (Figure 3B, 3C; Supplementary Table S7). Gene expression analysis showed that these genes were significantly down-regulated with the development of male PGCs (p<0.01, p<0.01) (Figure 3D, 3E; Supplementary Table S8, S9), and among these genes, we noticed that the expression of cytokines involved in proliferation regulation such as *ACVRL1*, *TGFB3*, *VEGFA*, *AGTR2* were significantly down-regulated (Figure 3F, 3G; Supplementary Table S8, S9). These preliminarily indicated that the proliferation capacity of PGCs is significantly down-regulated. To further identify the changes in proliferation capacity of PGCs during development, 105 and 314 DEGs were enriched for KEGG pathway analysis respectively. The results showed that the key signaling pathways involved in the regulation of cell proliferation during the whole development, such as the Cytokine-cytokine receptor interaction, MAPK signaling pathway, Wnt signaling pathway, and Notch signaling pathway, were significantly enriched, and gene expression analysis showed that these signaling pathways were inhibited to varying degrees with development progressed, consistent with the results of GO analysis (Figure 3H; Supplementary Table S8, S9). Similar characterization of changes in proliferation capacity could also be observed during the development of female PGCs (Supplementary Figure S4). We therefore concluded that PGCs experience loss of proliferation capacity during development (E3.5 to E5.5) *in vivo*.

### Early (E3.5) female and male PGCs are more suitable for *in vitro* long-term culture and cell line establishment

**Figure 4:**
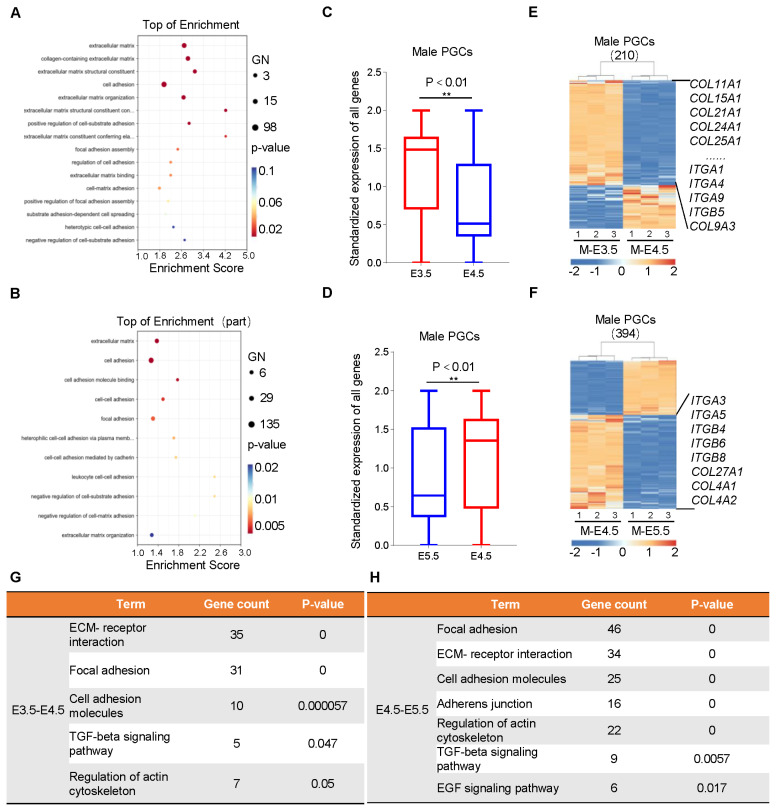
Changes in cell adhesion and intercellular communication during the development of male PGCs. (A, B) GO analysis of DEGs during male PGC formation to screen terms related to cell adhesion and intercellular communication. (C, D) Expression analysis of genes related to cell adhesion and intercellular communication during the development of male PGCs from E3.5 to E4.5 (C) and from E4.5 to E5.5 (D). (E, F) Specific expression analysis of genes related to cell adhesion and intercellular communication during the development of male PGCs from E3.5 to E4.5 (E) and from E4.5 to E5.5 (F). (G, H) Signaling pathway enrichment analysis of DEGs involved in cell adhesion and intercellular communication during the development of male PGCs from E3.5 to E4.5 (G) and from E4.5 to E5.5 (H). PGCs, primordial germ cells; GO, gene ontology; DEGs, differentially expressed genes. * p<0.05, ** p<0.01.

Long-term culture or cell line establishment of PGCs *in vitro* has always been a key difficulty restricting the specific application of PGCs, and the influencing factors not only involve cell proliferation, but also intercellular adhesion as well as information communication in the extracellular matrix. We found that during the development of male PGCs from E3.5 to E4.5 210 DEGs were significantly enriched in 16 GO terms related to cell adhesion and extracellular matrix interactions, and these DEGs were also significantly enriched in signaling pathways related to cell adhesion and extracellular matrix (Figure 4A, 4G). In gene expression analysis, we noticed that the adhesion between PGCs gradually decreased with development, which was confirmed by many down-regulated expressions of members of the collagen and integrin family members (Figure 4C, 4E). During further development of male PGCs we found that the decrease of cell adhesion and extracellular matrix interactions showed an obvious trend of enhancement (394 DEGs), and members of collagen family as well as integrin family were down-regulated to varying degrees similar to the development process before (Figure 3B, 3D, 3F, 3H). Interestingly, signaling pathway enrichment analysis of genes related to cell adhesion throughout the process revealed that signals such as transforming growth factor and vascular endothelial growth factor signaling pathway were enriched to varying degrees, suggesting that these signaling pathways were also involved in the regulation of cell adhesion during the development of PGCs. As is known to all, cell adhesion affects cell proliferation and motor capacity, and the decrease of adhesion performance during PGC development is consistent with the decrease of cell proliferation and migration capacity [24–27]. Similar phenomenon was observed during the development of female PGCs (Supplementary Figure S5). Taken together, we concluded that early PGCs are more suitable for long-term culture and cell line establishment *in vitro*.

During the comparison of differences between female and male PGCs, we found though with the progress of development the differences between female and male PGCs gradually increased, only a small number of DEGs between female and male PGCs at E3.5 and E4.5 were enriched in related terms such as cell proliferation, germline transmission ability and cell adhesion (Supplementary Figure S6A). Thus, their difference could not be assessed accurately. However, we noticed that at E5.5, female and male PGCs showed different characteristics. Gene expression characteristics showed that male PGCs had better proliferation and germline transmission ability than female PGCs (Supplementary Figure S6A, 6B). Differences in the current culture systems as well as *in vitro* expansion ability of male and female PGCs can also be explained by these results.

### Female and male PGCs at E5.5 are suitable cell materials for *in vitro* long-term cryopreservation

**Figure 5:**
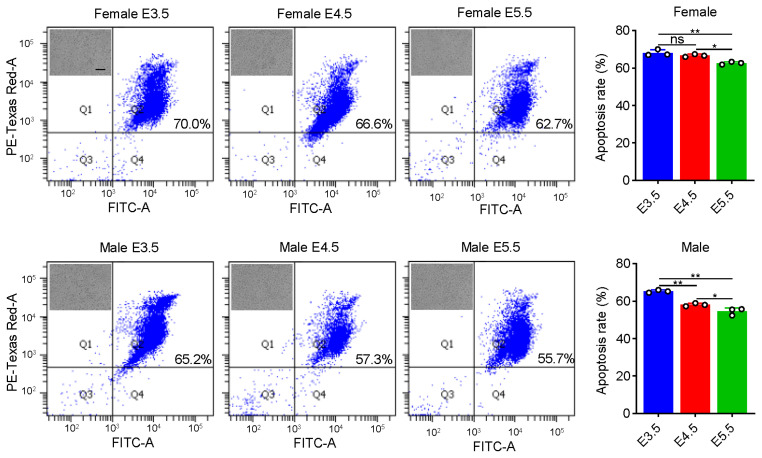
Cell apoptosis detection of female and male primordial germ cells at different developmental time points after cryopreservation and thawing. * p<0.05, ** p<0.01.

**Figure 6:**
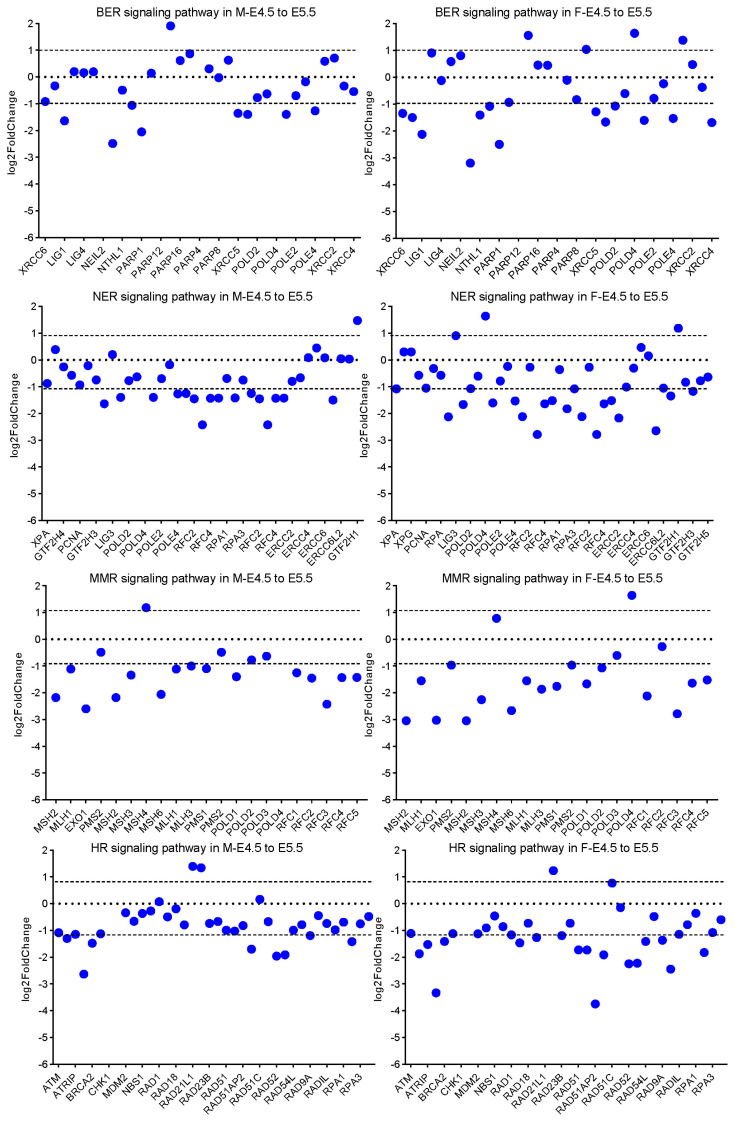
Analysis of signaling pathways related to damage repair in female and male primordial germ cells from E4.5 to E5.5 during development.

Long-term effective cryopreservation and thawing of PGCs is the key to the application of PGCs in the field of species resource conservation. Currently, the cryopreservation and recovery system for PGCs is immature, so we intended to provide a solution for optimizing the cryopreservation and recovery system from the gene expression level. PGCs at E3.5, E4.5, and E5.5 were frozen respectively and thawed after 30 days, followed by examining their apoptosis levels. The results showed that although PGCs at E5.5 also presented a large amount of apoptosis, their apoptosis levels were significantly lower than those of PGCs at E3.5 and E4.5 (Figure 5). Cell freezing can cause DNA damage, so we focused on different DNA damage repair pathways. The analysis showed that there were no obvious differences in the expression of genes related to repair pathways such as base excision repair (BER), nucleotide excision repair (NER), mismatch repair (MMR) and homologous recombination (HR) during PGC development (Supplementary Figure S8). However, during the development of female and male PGCs we noticed that from E3.5 to E4.5 the expression of DEGs in these pathways fluctuated but the differences were not significant, suggesting that these pathways were not activated in this process (Supplementary Figure S7). Interestingly, during further development of both female and male PGCs, we noted that genes related to BER, NER, MMR and HR pathways were significantly down-regulated, especially *XRCC* family and *POLD* family, indicating that compared with female and male PGCs at E3.5 and E4.5, female and male PGCs at E5.5 might possess the higher recovery efficiency after cryopreservation (Figure 6). Based on these results, we concluded that PGCs at late embryonic stage (E5.5) are suitable cell materials for cryopreservation and thawing.

## DISCUSSION

In recent years, injecting donor PGCs into the dorsal aorta, where donor PGCs possessing germline ability will migrate to the gonads through the blood circulation system and develop into functional gametes eventually in the bipotential gonads, has been the main method in studies on chimeric chickens. The researchers successfully obtained chimeric chickens by PGCs at E2.5 (HH14) from blood [5,7], at E5.5 (HH28) [28,29] or at E7 (HH27–28) [30] from embryonic gonads, but the efficiency was unstable, and the germline transmission ability of donor PGCs was lost to varying degrees during production of the next generation. On one hand, donor PGCs could not survive the competition because of incomplete clearance of endogenous PGCs. On the other hand, according to our results of the migration experiment and RNA-seq analysis, we found that the germline transmission ability of PGCs gradually decreases as development progressed (E3.5–E5.5), which is accompanied by a decline in migration ability. Therefore, E3.5 may be the preferred time point for allotransplantation, but it could be earlier. What is interesting is that male PGCs begin to prepare for spermatogenesis during E3.5–E4.5. This may explain why the success rate of producing chimeric chickens by transplanting male PGCs into male embryos is higher than by transplanting male PGCs into female embryos [5].

PGCs can be identified as early as Stage X in blastocysts, but this time point is not preferred for cell line establishment due to insufficient isolable PGCs. After gastrulation, PGCs appear in the germinal crescents and subsequently migrate through the blood circulation system to the embryonic gonads and colonize. In past studies on culturing PGCs, researchers mainly selected PGCs from germinal crescents at 30 h (HH6–8) [31], embryonic blood at E2.5–3 (HH14–18) [32–34] or embryonic gonads at E5–7 (HH26–31) [30,34] as the sources of PGCs for culture. Typically, the success rate of cell line establishment of PGCs from germinal crescents is higher than from blood or embryonic gonads. The reason for this, not to be overlooked, is that the density of PGCs in blood is not as high as that in germinal crescents and embryonic gonads, and thus the number of PGCs obtained from embryonic blood is limited. In this study, we found a gradual decline in the proliferation capacity of PGCs with the progression of development (E3.5–E5.5), accompanied by a decrease in cell adhesion and extracellular matrix information exchange, which also provides a rational explanation for the superior performance of the germinal crescents as a source of isolating and culturing PGCs. Considering the difficulty of separating germinal crescents, it is likely that embryonic gonads at around E3.5 are the better choice for culturing PGCs in quantity. It is worth noting that female PGCs do not perform as well as male PGCs in long-term culture *in vitro*. Tonus et al [33] found that female PGCs faded during culture. In the study of Liu et al [7], female PGCs proliferated more slowly than male PGCs, and during proliferation female PGCS would form cell clusters to hinder cell division. This may be explained by our finding that male PGCs possess better cell adhesion than female PGCs and male PGCs communicate more closely with each other. Since ZW chromosomes store complete genetic information, it is necessary to establish and optimize culture system for female PGCs for germplasm conservation. Consequently, researchers have made many efforts for the culture of female PGCs. For example, Woodcock et al [8] replaced chicken serum in culture medium with ovotransferrin, which increased the success rate of primary culture of female PGCs by 31%.

During cryopreservation and thawing, cells undergo stress caused by factors including changes in osmotic pressure, temperature and pH value which cause massive apoptosis after thawing. For a long time, researchers have been trying to improve the recovery efficiency of cells by optimizing the cryopreservation and thawing system [35–39]. However, we attempted to reduce apoptosis after thawing and cryopreservation by selecting appropriate cell materials from the perspective of the cells themselves. DNA damage can lead to apoptosis, but in fact cells have their own DNA repair mechanisms that respond to DNA damage caused by cryopreservation and thawing to participate in maintaining genome stability. Based on this, we found from the transcription level that the genes related to the repair pathways were significantly down-regulated during the development from E4.5 to E5.5, indicating that PGCs at E5.5 have stronger DNA damage repair ability. Therefore, PGCs at E5.5 are more suitable for cryopreservation.

## CONCLUSION

In this study, based on RNA-seq we found that female and male PGCs at E3.5 are more suitable for allotransplantation *in vitro* because of their stronger germline transmission ability; the development process of female and male PGCs is accompanied by the gradual loss of proliferation capacity, and PGCs at E3.5 are more suitable for *in vitro* long-term culture and cell line establishment; female and male PGCs at E5.5 are suitable cell materials for *in vitro* long-term cryopreservation. To sum up, in view of the different characteristics of female and male PGCs at different developmental time points, we provide a theoretical basis for selecting PGCs as cell materials in the specific application.

## Data Availability

Data and code related to this paper may be requested from the authors. The data of RNA-seq for PGC in this paper has been submitted to SRA with the Accession No. PRJNA 1062092.

## SUPPLEMENTARY MATERIAL

**Supplementary Table S1**. GO terms related to germline transmission ability during the development of male PGCs

**Supplementary Table S2**. Genes related to germline transmission ability during the development of male PGCs from E3.5 to E4.5

**Supplementary Table S3**. Genes related to germline transmission ability during the development of male PGCs from E4.5 to E5.5

**Supplementary Table S4**. GO terms related to germline transmission ability during the development of female PGCs

**Supplementary Table S5**. Genes related to germline transmission ability during the development of female PGCs from E3.5 to E4.5

**Supplementary Table S6**. Genes related to germline transmission ability during the development of female PGCs from E4.5 to E5.5

**Supplementary Table S7**. GO terms related to cell proliferation during the development of male PGCs

**Supplementary Table S8**. Genes related to cell proliferation during the development of male PGCs from E3.5 to E4.5

**Supplementary Table S9**. Genes related to cell proliferation during the development of male PGCs from E4.5 to E5.5

**Supplementary Table S10**. qRT-PCR primers sequence of related genes

**Supplementary Figure S1**. Isolation and identification of chicken PGCs.

**Supplementary Figure S2**. Large differences were found among PGCs of E3.5, 4.5, and 5.5, but not between males and females.

**Supplementary Figure S3**. Evaluation of the migration ability of female PGCs during development.

**Supplementary Figure S4**. Changes in the proliferation ability of female PGCs during development.

**Supplementary Figure S5**. Changes in cell adhesion and intercellular communication during the development of female PGCs.

**Supplementary Figure S6**. Comparison of differences between female and male PGCs at different developmental time points.

**Supplementary Figure S7**. Analysis of signaling pathways related to damage repair in female and male PGCs from E3.5 to E4.5 during development.

**Supplementary Figure S8**. Analysis of signaling pathways related to damage repair among female and male PGCs at different developmental time points.
