# Supplementary material for: Comparison of primordial germ cell differences at different developmental time points in chickens
Source: Anim Biosci. 2024 Aug 5;37(11):1873–86. doi: 10.5713/ab.24.0283 (PMC11541041; doi:10.5713/ab.24.0283)
Supplement: Supplementary file 1 [file ab-24-0283-Supplementary-Table-1.pdf]

Table S1. GO terms related to germline transmission ability during the development of male PGCs

	Term	ListHits	p-value	Enrichment_score
E3.5-4.5	male gonad development	13	0.012822	1.914154
	negative regulation of endothelial cell migration	5	0.02935	2.638097
	spermatid differentiation	3	0.03063	3.79886
	substrate-dependent cell migration	3	0.03063	3.79886
	leukocyte migration	11	0.034956	1.785789
	negative regulation of blood vessel endothelial cell migration	4	0.040215	2.81397
	positive regulation of leukocyte migration	3	0.054142	3.165717
	regulation of cell migration	13	0.065341	1.552993
	retinoic acid binding	3	0.083845	2.713472
	positive regulation of endothelial cell migration	6	0.1008	1.808981
	male meiosis I	4	0.107185	2.110478
	positive regulation of blood vessel endothelial cell migration	4	0.107185	2.110478
	negative regulation of cell migration involved in sprouting angiogenesis	4	0.107185	2.110478
E4.5-5.5	leukocyte migration	24	1.786768	0.000488
	male gonad development	24	1.620557	0.003244
	positive regulation of cell migration	56	1.354966	0.003629
	negative regulation of retinoic acid receptor signaling pathway	5	2.419582	0.020709
	positive regulation of vascular associated smooth muscle cell migration	7	2.032449	0.023697
	positive regulation of endothelial cell migration	12	1.659142	0.027492
	regulation of cell migration	25	1.369575	0.037281
	substrate-dependent cell migration, cell extension	4	2.322799	0.050939
	mesenchyme migration	4	2.322799	0.050939
	negative regulation of vascular associated smooth muscle cell migration	4	2.322799	0.050939
	negative regulation of neuron migration	4	2.322799	0.050939
	cellular response to gonadotropin-releasing hormone	4	2.322799	0.050939

	cell migration involved in sprouting angiogenesis	6	1.742099	0.088408
