# Supplementary material for: Comparison of primordial germ cell differences at different developmental time points in chickens
Source: Anim Biosci. 2024 Aug 5;37(11):1873–86. doi: 10.5713/ab.24.0283 (PMC11541041; doi:10.5713/ab.24.0283)
Supplement: Supplementary file 2 [file ab-24-0283-Supplementary-Table-2.pdf]

Table S2. Genes related to germline transmission ability during the development of male PGCs from E3.5 to E4.5

| Gene Id | Expression Male3.5-2 | Expression Male3.5-1 | Expression Male3.5-3 | Expression Male4.5-1 | Expression Male4.5-2 | Expression Male4.5-3 |
|---|---|---|---|---|---|---|
| *ACVRL1* | 5.863852 | 6.198249 | 5.622993 | 1.725056 | 1.982807 | 1.204948 |
| *AGTR2* | 2.741962 | 3.237265 | 2.98483 | 0.212775 | 0.182871 | 0.359562 |
| *MEF2C* | 4.885858 | 5.113514 | 4.663016 | 1.21602 | 1.16148 | 1.340015 |
| *RGCC* | 284.2051 | 285.2532 | 291.666 | 18.73324 | 17.86941 | 16.45824 |
| *ADD2* | 5.68859 | 5.497193 | 6.043371 | 21.7856 | 19.44484 | 19.44632 |
| *CD244* | 4.589633 | 4.869742 | 4.627188 | 1.063857 | 1.192507 | 0.792334 |
| *CD34* | 7.161522 | 7.91175 | 8.019867 | 0.316192 | 0.199011 | 0.189821 |
| *CD74* | 62.98821 | 59.50873 | 59.83745 | 17.5103 | 16.45058 | 17.1194 |
| *DOK2* | 4.571171 | 4.220002 | 4.243225 | 1.483791 | 1.591867 | 1.253044 |
| *INPP5D* | 5.334344 | 5.908634 | 6.234418 | 1.368759 | 1.421466 | 1.704692 |
| *ITGA4* | 8.700017 | 8.321839 | 8.414447 | 3.05135 | 3.454886 | 3.260432 |
| *JAML* | 0.114366 | 0.080649 | 0.04359 | 0.922885 | 0.590543 | 1.040038 |
| *LOC121107557* | 3.334889 | 2.779268 | 3.35782 | 1.341699 | 0.843868 | 0.72904 |
| *MADCAM1* | 16.73713 | 17.13386 | 17.84917 | 2.626899 | 2.049893 | 1.980766 |
| *VSIG10L* | 47.41154 | 46.68765 | 47.68939 | 13.10801 | 14.26049 | 14.94722 |
| *EPHA8* | 0.031878 | 0.030667 | 0.022816 | 0.097585 | 0.109138 | 0.088053 |
| *FMNL1* | 2.726105 | 3.137173 | 2.724502 | 1.087865 | 1.359536 | 1.057399 |
| *VEGFC* | 8.668518 | 9.288697 | 9.509763 | 3.609585 | 2.698394 | 3.456123 |
| *LOC107049412* | 0.653049 | 0.532151 | 0.862877 | 1.291724 | 1.485193 | 2.308905 |
| *RHBDD1* | 3.211447 | 3.14181 | 3.151016 | 8.661589 | 9.082453 | 9.58663 |
| *TMEM119* | 3.342583 | 3.494318 | 3.399595 | 0.792272 | 0.898836 | 0.888568 |
| *ACVRL1* | 5.863852 | 6.198249 | 5.622993 | 1.725056 | 1.982807 | 1.204948 |
| *ADAMTS9* | 10.8169 | 11.52872 | 11.71309 | 2.725489 | 2.374109 | 2.695093 |
| *APOH* | 0.459193 | 0.425209 | 0.289578 | 2.167485 | 1.975322 | 2.167759 |
| *DLL4* | 6.702903 | 6.313609 | 6.947604 | 1.166416 | 1.059915 | 0.970068 |
| *STC1* | 6.442871 | 6.88083 | 6.965174 | 2.186052 | 2.255907 | 2.602932 |
| *AHSG* | 0.219002 | 0.527265 | 0.394594 | 0.084387 | 0 | 0.021997 |
| *COL9A3* | 5.151878 | 5.663808 | 4.986897 | 1.520529 | 1.381695 | 1.409273 |
| *HOXA10* | 0.976811 | 1.721028 | 1.177295 | 2.741522 | 2.498467 | 2.187661 |
| *HOXA11* | 0.079006 | 0.024142 | 0.070464 | 0.192551 | 0.208659 | 0.235686 |
| *KITLG* | 5.999106 | 5.0032 | 5.925743 | 1.172627 | 1.858719 | 1.565889 |
| *MAS1* | 0.063064 | 0.064236 | 0.012499 | 0.147014 | 0.152675 | 0.153289 |
| *MRGPRH* | 16.12992 | 16.38151 | 17.2284 | 3.200016 | 3.622983 | 3.179591 |
| *NR0B1* | 0.647513 | 0.510619 | 0.972856 | 18.43693 | 16.94028 | 17.83924 |
| *NR5A1* | 0.11949 | 0.041969 | 0.142911 | 0.196473 | 0.204039 | 0.172992 |
| *RHOBTB3* | 3.990473 | 4.033939 | 4.004276 | 8.989455 | 8.494965 | 9.106632 |
| *SRD5A2* | 0.454407 | 0.490081 | 0.30462 | 0.920527 | 1.147169 | 1.033652 |
| *TRAIL-LIKE* | 21.66505 | 19.38234 | 19.28779 | 4.626705 | 5.853385 | 5.263687 |
| *YBX3* | 242.1315 | 233.6583 | 230.3592 | 644.0434 | 635.0569 | 652.8487 |
| *DOCK10* | 7.379314 | 7.773267 | 8.376881 | 1.741497 | 1.743709 | 2.343943 |

| | | | | | | |
|---|---|---|---|---|---|---|
| *FRMD5* | 0.56051 | 0.356831 | 0.81235 | 1.536813 | 1.696222 | 2.004949 |
| *LAMA1* | 10.07579 | 9.725665 | 10.32356 | 2.436481 | 2.032159 | 2.884953 |
| *LAMA4* | 2.95612 | 3.098819 | 3.046268 | 1.004102 | 0.668568 | 0.921727 |
| *LAMA5* | 63.69037 | 66.96448 | 68.38629 | 20.45913 | 20.63088 | 20.811 |
| *PAX6* | 0.295498 | 0.205555 | 0.335685 | 0.030548 | 0.087243 | 0.023889 |
| *PLXND1* | 14.58771 | 16.18084 | 16.03066 | 4.59013 | 4.710358 | 4.17314 |
| *RHOD* | 0.390653 | 0.066319 | 0.064521 | 0.551934 | 0.465714 | 0.575493 |
| *RND2* | 0.795504 | 1.207794 | 1.085809 | 2.194839 | 1.965531 | 2.935282 |
| *ROBO4* | 0.796268 | 0.803346 | 1.074657 | 0.072322 | 0.041726 | 0.008379 |
| *SERPINE2* | 457.7954 | 447.7083 | 445.4767 | 181.5001 | 184.8682 | 183.6849 |
| *SPATA13* | 16.91846 | 17.49804 | 17.51432 | 6.258287 | 6.768186 | 6.48685 |
| *TMIGD1* | 0.032049 | 0.032645 | 0.04764 | 0.101881 | 0.08817 | 0.300985 |
| *ITGA2B* | 1.533066 | 1.390254 | 1.916671 | 2.899411 | 2.868692 | 2.851644 |
| *ADGRA2* | 2.816414 | 2.557792 | 2.828823 | 0.93009 | 0.722329 | 0.674637 |
| *ETS1* | 25.06761 | 25.91528 | 25.36594 | 6.829239 | 6.334542 | 5.449232 |
| *NRP2* | 100.0336 | 103.2791 | 103.404 | 40.42377 | 41.96202 | 43.26562 |
| *SASH1* | 20.26032 | 20.69582 | 20.64213 | 5.938055 | 6.181856 | 5.838761 |
| *TEK* | 1.426397 | 1.310851 | 1.281599 | 0.14107 | 0.293004 | 0.252157 |
| *WNT7A* | 0.046476 | 0.639095 | 0.552685 | 0.886466 | 1.329757 | 0.924304 |
| *CRABP1* | 9.968732 | 8.95281 | 10.09854 | 3.556681 | 3.527258 | 3.508038 |
| *CRABP2* | 12.68603 | 11.13345 | 12.40003 | 4.192618 | 3.700954 | 4.262289 |
| *LOC121111296* | 0.705718 | 0.878579 | 0.984272 | 2.04954 | 1.121757 | 1.963752 |
| *FGF2* | 46.03171 | 47.8621 | 51.45571 | 9.179021 | 8.645736 | 8.958738 |
| *MAP2K3* | 16.89457 | 15.12181 | 17.23478 | 29.00412 | 28.23117 | 28.72755 |
| *NFE2L2* | 27.41042 | 26.98144 | 26.73344 | 51.616 | 53.59815 | 53.65045 |
| *MEIOC* | 0.26767 | 0.069909 | 0.163234 | 0.472721 | 0.679741 | 0.37157 |
| *MOV10L1* | 5.401737 | 5.194076 | 5.452911 | 12.43173 | 12.67103 | 12.56371 |
| *REC8* | 1.458003 | 1.250054 | 1.070643 | 3.145481 | 2.527867 | 2.816177 |
| *TDRD9* | 7.64086 | 7.498897 | 7.054587 | 15.18729 | 12.74303 | 13.77746 |
| *CARD10* | 1.235624 | 0.962454 | 0.857788 | 2.13551 | 2.239556 | 2.460281 |
| *MEOX2* | 6.03211 | 5.148722 | 6.885692 | 1.537279 | 1.193155 | 0.961739 |
| *MMRN2* | 2.704378 | 2.934748 | 2.771568 | 0.110709 | 0.150348 | 0.088796 |
