# Supplementary material for: Comparison of primordial germ cell differences at different developmental time points in chickens
Source: Anim Biosci. 2024 Aug 5;37(11):1873–86. doi: 10.5713/ab.24.0283 (PMC11541041; doi:10.5713/ab.24.0283)
Supplement: Supplementary file 3 [file ab-24-0283-Supplementary-Table-3.pdf]

Table S3. Genes related to germline transmission ability during the development of male PGCs from E4.5 to E5.5

gene_id	Expression Male5.5-2	Expression Male5.5-1	Expression Male5.5-3	Expression Male4.5-1	Expression Male4.5-2	Expression Male4.5-3
ADGRG3	0.012261	0.012635	0.013159	0.128008	0.132937	0.133472
AMOTL1	4.165573	4.012257	4.317118	10.29712	8.950161	9.380934
AMOTL2	7.375858	7.143115	6.613067	24.18988	24.19785	25.67616
DOCK10	7.941493	8.337605	8.191646	1.741497	1.743709	2.343943
LAMA1	14.1666	14.26914	14.27706	2.436481	2.032159	2.884953
LAMA3	0.427169	0.52601	0.461348	23.71824	23.85963	24.24515
LAMA4	0.294508	0.379354	0.400044	1.004102	0.668568	0.921727
LOC107056377	0.17259	0.076221	0.63509	0	0.026733	0
LOC121109863	12.60943	12.94781	11.65627	1.837474	1.702913	2.194803
NEXN	0.710665	0.70617	0.417686	2.261209	1.981362	2.016963
PAX6	1.294749	1.515118	0.997061	0.030548	0.087243	0.023889
PLXND1	2.248661	2.116631	1.980706	4.59013	4.710358	4.17314
RHOB	23.35636	22.39353	22.03885	67.17013	77.13252	70.84639
RHOC	7.250771	6.975287	6.308316	33.14728	35.57288	32.49053
RHOD	0	0	0	0.551934	0.465714	0.575493
RHOF	0.687127	0.920489	1.179948	1.537252	1.894451	1.239552
RND2	0.655054	0.470943	0.376047	2.194839	1.965531	2.935282
RND3	69.34413	69.38767	71.39961	27.39341	25.51791	25.66636
ROBO4	0.007697	0	0	0.072322	0.041726	0.008379
SLK	52.15416	54.08749	54.39595	14.75519	15.28349	15.24772
SRCIN1	4.870539	5.958199	5.427766	1.196084	1.351945	1.28159
SST1	9.117539	8.638433	6.771018	20.61704	23.70664	21.63819
TMIGD1	0.065056	0	0	0.101881	0.08817	0.300985
TMSB4X	693.1601	715.1807	678.831	1669.612	1618.008	1631.95
VCL	42.27317	41.13115	42.22955	95.66645	95.7671	98.28133
BCAR1	19.15654	18.07102	18.35713	43.97391	44.303	42.31537
BMPR2	10.88997	10.65465	11.53432	18.43178	18.91335	19.72375
FGF1	0.219191	0.387208	0.134428	0.997103	1.001548	1.295319
GATA3	0.187753	0.141007	0.075139	0.39204	0.424388	0.336027
ITGB1BP1	5.061867	4.943752	5.248312	9.865813	9.887474	9.33737
NRP2	21.60672	22.19546	22.0055	40.42377	41.96202	43.26562
PRKCA	14.72875	16.05946	14.1996	3.773895	3.422577	3.471134
PTK2B	0.117991	0.162115	0.105529	2.740894	2.841107	2.51537
TEK	0.04504	0.033152	0.027623	0.14107	0.293004	0.252157
WNT7A	0.047171	0.072913	0.531581	0.886466	1.329757	0.924304
ZC3H12A	7.568283	8.01709	8.349964	14.93282	15.51501	15.23894
AGO4	58.87713	57.62611	57.26869	18.87169	17.85867	20.06935
BCL2L1	14.76366	14.56015	13.85589	27.69555	25.27144	26.47051
COL9A3	3.7927	3.835333	3.690643	1.520529	1.381695	1.409273
HOXA10	0.214359	0.19328	0.316337	2.741522	2.498467	2.187661

HOXA11	0.008019	0	0	0.192551	0.208659	0.235686
HOXA9	0.391431	0.596272	0.675827	6.432075	7.10416	7.373584
INHBA	1.24909	1.322369	0.900526	10.86084	11.20907	12.20476
KIT	30.88211	32.05111	31.01531	5.491099	5.576382	5.947158
LOC430303	1.054346	1.247277	1.154221	0.145302	0.137179	0.174459
LOC776507	0.245355	0	0.153609	1.174061	1.795653	1.135144
LRP2	46.68188	49.0447	47.87015	5.96139	6.503743	7.296691
MRGPRH	0.048079	0.136247	0.090303	3.200016	3.622983	3.179591
NASP	154.1975	154.5584	155.7239	37.66096	35.72299	37.33243
NCOA1	16.19517	16.21951	15.77721	6.050218	6.073599	6.528262
NR0B1	1.589974	1.288899	0.72809	18.43693	16.94028	17.83924
PATZ1	13.65989	13.74267	13.9524	3.195445	3.281015	3.355788
PDGFA	5.01025	6.649247	3.381189	27.66023	26.17381	21.27946
RHOBTB3	0.295852	0.226023	0.312052	8.989455	8.494965	9.106632
RRM1	39.65323	40.7303	38.29701	13.32341	12.4353	12.42979
SIX5	6.602611	7.503258	6.964923	15.52678	15.29881	16.18132
SOX9	5.707052	6.655281	6.125164	17.83772	20.61953	17.9759
TGFB2	4.16766	3.640564	4.011931	15.23583	16.14925	16.66093
YBX3	191.4446	191.3221	188.5143	644.0434	635.0569	652.8487
CLEC14A	3.131941	3.72239	3.093308	6.64001	5.604056	7.028021
EFNB2	70.18839	74.98281	70.33366	6.847942	9.207029	9.371572
EGR3	4.442445	5.035618	4.247773	1.075188	1.357423	1.450812
GREM1	1.783698	2.507861	1.890041	0.078909	0.204868	0.131643
MIA3	8.510328	8.43754	8.841784	16.29295	17.02927	17.94082
MMRN2	0.028549	0.025216	0.035018	0.110709	0.150348	0.088796
ADGRG1	14.54769	15.64952	13.13316	26.34045	28.59027	24.76353
GNRH1	0	0	0	0.470061	0.08136	0.735188
KIAA2022	11.11355	11.23142	11.04313	2.314849	2.36824	2.165413
NRG3	3.872738	3.984809	3.796651	0.518987	0.601643	0.799125
DRD4	2.46245	2.178417	2.867249	0.897384	0.327438	0.354044
MEF2C	0.607686	0.734588	0.522601	1.21602	1.16148	1.340015
NFE2L2	11.5521	11.36822	11.89391	51.616	53.59815	53.65045
PRKACA	4.784833	4.245844	4.124947	1.167936	1.188892	0.771669
ACTBL2	0.718675	1.00753	0.932767	0.139595	0.262758	0.236524
ACTC1	1.849716	1.906089	1.414762	9.855614	10.0046	10.20686
ACTG2	12.2025	13.04097	11.7844	204.4754	211.8582	206.5
TGFB3	30.8429	28.22994	28.20698	64.43011	65.36925	67.74986
MYH10	62.64092	63.17107	63.2362	23.41082	24.07049	24.18813
NTN1	2.446186	2.535059	2.610482	0.464346	0.241113	0.257213
P2RY12	0.298434	0.193304	0.20133	1.175084	1.044679	1.327346
SHTN1	24.7526	25.34341	26.36324	9.800447	9.579619	9.914352
ANXA5	45.5246	48.42002	46.16959	99.89916	99.3473	94.39999
CRHBP	1.895075	1.893951	2.187222	0.019885	0.02065	0.082934
LOC101749220	0.284673	0.664925	0.325898	0.138697	0.123461	0.082639

UMODL1	0.017082	0.077452	0.121002	0.774012	0.633422	0.795893
ARHGEF39	10.89695	13.22627	12.40342	5.155614	4.839965	4.848213
ATP8A1	57.75198	59.05374	58.68359	8.477194	8.927077	9.691183
CCLI7	0.213384	0	0.03817	1.485203	0.424158	1.355024
CCLI8	1.006593	1.075688	0.400125	1.712607	2.223193	1.501619
CD274	0.659046	0.979213	0.827959	2.22955	2.392953	2.285787
CDH13	9.342719	9.35922	9.088796	2.378952	2.269382	2.144823
CSF1	3.279036	3.27532	3.594833	6.563317	7.317712	5.299584
CSF1R	1.644756	1.858903	1.978794	6.813336	6.788067	6.837033
EDN1	1.000071	0.712292	0.505101	16.0455	16.91849	15.01733
FAM110C	1.931831	1.622057	1.678436	9.300143	8.588818	9.513523
FAM65A	2.712043	2.827068	2.669109	7.330109	8.435482	8.138847
GRB7	0	0.028138	0.146533	6.172076	6.187698	7.119212
GTSE1	21.82902	23.05397	20.71212	6.623529	6.37741	6.566601
HOMER3	3.798609	3.797114	3.45461	6.975647	6.835925	6.769041
IL1B	1.267929	0.916892	1.289199	6.665257	5.305999	4.770394
ITGA5	8.043026	9.483749	9.508168	59.4579	55.56417	56.04458
LEF1	10.80179	10.6343	10.33303	2.658069	2.505822	2.74462
LGR6	0.546831	0.70599	0.688082	2.001469	2.133058	2.35375
LOC101751416	0.043677	0.084391	0.041018	0.205203	0.071035	0.124811
LOC121106433	3.690321	5.399021	4.54745	38.29039	40.16002	40.12319
LYN	3.0538	3.349893	3.494547	9.15884	9.831943	9.651376
MAPK8	27.73424	28.77419	28.03426	6.056234	6.654106	5.491975
MCAM	21.34989	21.55275	21.78563	38.87216	35.0797	33.4964
MIEN1	15.58705	17.62851	16.02979	33.17661	30.16373	30.62653
MYO1F	0.645455	0.804721	0.906553	3.244596	3.931125	3.695376
NTRK3	13.35584	13.38642	13.72934	0.870101	0.994895	0.997343
ONECUT1	1.307335	0.846797	1.262802	0.341226	0.323991	0.396453
PDCD1LG2	1.160079	0.996195	0.622535	3.818526	3.598707	3.735962
PDGFB	0.79178	0.504214	0.525149	24.0563	23.42967	22.06154
PDPN	5.906906	5.219748	5.575427	13.73639	12.10711	13.05429
PIK3R1	1.352957	1.390661	1.356498	2.581897	2.614471	2.725663
PODXL	6.482297	6.796635	6.878147	23.07185	23.50564	23.61254
PTP4A1	67.91703	68.94998	66.85629	143.6844	141.0058	141.6307
SEMA3A	24.48878	23.90103	25.16008	8.555749	7.604413	7.540101
SEMA3C	0.668687	0.834379	0.70303	6.511148	6.322918	6.44244
SEMA3D	4.885335	4.311921	4.361773	9.557814	10.52463	9.780807
SEMA3DL	1.135882	0.951667	1.054786	1.804631	1.611744	1.908542
SEMA3G	1.895872	1.930113	1.618008	0.798896	0.606766	0.397849
SEMA6B	10.02205	9.881746	10.45728	3.245403	2.964975	2.729492
SEMA6D	4.990261	5.053838	5.429606	1.64281	1.614806	1.561462
SPHK1	14.49774	15.86353	17.60294	32.2597	32.57464	31.09893
TBX20	2.954022	2.128547	2.979738	0.463775	1.035511	1.039677
TGFBR2	1.171738	1.559334	1.056369	12.45705	11.20167	12.24531

VILL	0.8799	0.906716	0.731549	20.04201	18.94604	20.64791
VSIR	1.025312	0.946678	0.801112	6.011709	5.762956	6.036158
WNT5B	5.068524	3.739468	5.197233	33.05458	33.605	32.56155
XBP1	64.97792	64.81932	68.17142	143.667	133.7319	141.4889
ZNF703	2.480123	2.386936	2.624156	7.816858	7.635864	7.220851
ADD2	7.825027	6.726853	8.41344	21.7856	19.44484	19.44632
CD244	0.485231	0.200008	0.121515	1.063857	1.192507	0.792334
CD74	8.611417	6.993449	6.821705	17.5103	16.45058	17.1194
DBH	0.260908	0.158872	0.127283	10.62349	11.43119	10.3798
DOK2	1.010115	0.689899	0.705938	1.483791	1.591867	1.253044
F11R	31.13497	33.89018	32.74409	114.0324	123.7631	112.8117
GRB14	0.555472	0.700824	0.40891	0.078068	0.127402	0.089152
INPP5D	0.449149	0.519128	0.325712	1.368759	1.421466	1.704692
ITGA4	18.21356	18.94988	19.12044	3.05135	3.454886	3.260432
JAM2	10.78501	9.086399	9.539623	3.428218	2.777592	2.311132
JAML	0.125004	0.174819	0.210827	0.922885	0.590543	1.040038
LOC107049116	0	0	0	0.083603	0.121552	0.122041
LOC107049666	2.661229	2.099779	2.449876	0.569633	0.434621	0.496977
LOC121107557	0.108601	0.354384	0.058279	1.341699	0.843868	0.72904
NKX2-3	2.641204	1.913078	2.711457	3.856514	4.378542	3.979459
PDE4B	4.936981	4.397111	5.204894	8.742677	9.126702	8.608539
PDE4C	0.375283	0.534378	0.446717	3.519154	4.26871	3.55795
PIK3CB	5.947158	5.302926	6.067255	11.03123	11.27461	10.65765
SELPLG	0.083164	0.222817	0.12496	0.642515	0.613154	0.506982
SLC7A10	3.11741	3.624706	3.345799	0.156643	0.234974	0.235919
TNFRSF10B	3.982076	4.506227	4.404911	35.73345	38.19544	38.66822
VSIG10L	3.844595	5.044051	3.702257	13.10801	14.26049	14.94722
CYP26C1	5.574662	5.973608	5.687262	0.575516	0.722997	0.735584
DHRS3	1.179102	1.05792	1.058208	15.5682	15.45132	14.48995
EZH2	35.7518	34.20788	33.57829	12.13309	11.79334	11.50889
FOXS1	0.387938	0.25248	0.394445	0.937941	0.951921	0.800163
ZNF536	0.140176	0.147658	0.173848	0.331723	0.324232	0.274671
ADAMTS1	2.38701	2.254778	1.700301	7.9288	6.878451	8.027497
DOCK4	3.573443	3.454928	3.552953	1.316195	1.520939	1.388832
DOCK5	2.288083	2.684108	2.630066	5.050767	5.719928	5.431213
IQGAP3	15.385	15.64342	15.50381	26.09961	27.44628	26.7817
NR4A3	0.143761	0.237926	0.25248	4.211659	4.416348	4.234934
