# Supplementary material for: Comparison of primordial germ cell differences at different developmental time points in chickens
Source: Anim Biosci. 2024 Aug 5;37(11):1873–86. doi: 10.5713/ab.24.0283 (PMC11541041; doi:10.5713/ab.24.0283)
Supplement: Supplementary file 4 [file ab-24-0283-Supplementary-Table-4.pdf]

Table S4. GO terms related to germline transmission ability during the development of female PGCs

	Term_description	ListHit	FoldEnrichment	p-value
E3.5-4.5	leukocyte migration	14	2.32376	0.001395
	retinol dehydrogenase activity	6	2.774284	0.013247
	retinol metabolic process	6	2.427499	0.026847
	cerebral cortex tangential migration	3	3.883998	0.028826
	negative regulation of vascular associated smooth muscle cell migration	3	3.883998	0.028826
	negative regulation of blood vessel endothelial cell migration	4	2.877036	0.037371
	positive regulation of leukocyte migration	3	3.236665	0.051098
	gonad development	3	3.236665	0.051098
	positive regulation of endothelial cell migration	6	1.849523	0.092597
	negative regulation of endothelial cell migration	4	2.157777	0.100471
	positive regulation of cell migration involved in sprouting angiogenesis	4	2.157777	0.100471
E4.5-5.5	positive regulation of cell migration	71	1.452959	3.26E-05
	neural crest cell migration	24	1.59289	0.002569
	leukocyte migration	25	1.57417	0.002674
	cellular response to retinoic acid	22	1.500708	0.010686
	positive regulation of endothelial cell migration	13	1.520198	0.040826
	blood vessel endothelial cell migration	5	2.046421	0.044353
	negative regulation of retinoic acid receptor signaling pathway	5	2.046421	0.044353
	neuron migration	41	1.243011	0.045058
	regulation of fibroblast migration	6	1.841779	0.054376
	cell migration involved in sprouting angiogenesis	7	1.718993	0.060255
	positive regulation of smooth muscle cell migration	8	1.637137	0.063538
	regulation of neuron migration	8	1.637137	0.063538
	germ cell development	9	1.578667	0.065133
	negative regulation of cell migration	34	1.227852	0.076179
	cell migration	64	1.155626	0.077918
	ameboidal-type cell migration	4	1.964564	0.09266
	regulation of epithelial cell migration	4	1.964564	0.09266

	negative regulation of vascular associated smooth muscle cell migration	4	1.964564	0.09266
	retinoic acid-responsive element binding	4	1.964564	0.09266
