# Supplementary material for: Comparison of primordial germ cell differences at different developmental time points in chickens
Source: Anim Biosci. 2024 Aug 5;37(11):1873–86. doi: 10.5713/ab.24.0283 (PMC11541041; doi:10.5713/ab.24.0283)
Supplement: Supplementary file 5 [file ab-24-0283-Supplementary-Table-5.pdf]

Table S5. Genes related to germline transmission ability during the development of female PGCs from E3.5 to E4.5

gene_id	Expression_ Female4.5-1	Expression_ Female4.5-3	Expression_ Female4.5-2	Expression_ Female3.5-1	Expression_ Female3.5-2	Expression_ Female3.5-3
ADD2	29.85846	31.9704	32.7862	7.304934	7.448395	7.785902
CD244	2.019317	2.224883	1.549119	3.795103	5.209984	4.689457
CD34	0.416645	0.215295	0.283542	9.373483	10.18775	10.90175
CD74	23.48225	24.96785	23.12138	66.63344	68.08781	69.9235
GRB7	10.36807	10.94804	11.20627	5.551763	5.873307	5.980368
INPP5D	2.880763	2.731244	3.087874	7.842463	8.024153	7.887265
ITGA4	4.307186	5.019494	5.039578	11.85728	10.86041	11.02706
JAM3	46.88374	48.4603	49.18222	20.88813	19.83219	21.5309
JAML	0.902848	0.914026	1.177014	0.104278	0.103053	0.17693
LOC107049116	0.198295	0.290321	0.065974	0.714155	0.470509	0.367462
LOC107050189	0.196366	0.067646	0.097998	0.505147	0.632335	0.363887
LOC121107557	1.456693	0.984333	1.155707	4.881117	4.519904	5.21004
MADCAM1	3.960139	3.895634	3.308552	17.20536	16.57065	16.27825
VSIG10L	16.51348	16.12094	15.7382	41.12513	39.53992	39.79449
ADIPOQ	1.010933	0.80629	1.360001	0.067842	0.234657	0.366528
MEF2C	1.635838	1.333366	1.275722	5.347574	5.60354	6.260476
NFE2L2	57.20251	59.29847	59.85549	28.31549	27.46331	28.47161
ARX	1.300901	1.008333	1.235337	0.399726	0.569577	0.639203
ESX1	1.34752	1.564317	1.050196	0.30391	0.225254	0.31719
RELN	0.311409	0.345574	0.450949	0.123447	0.197271	0.178024
ALDH1A1	12.66204	12.15386	12.88706	31.12058	31.67941	34.06784
ALDH1A2	17.47175	17.96894	16.58504	48.89114	47.88561	48.80503
LOC772005	0.584634	0.415389	0.638241	0.150396	0.074314	0.018467
RBP1	0.02095	0.082997	0.097584	0.025151	0.010652	0.028235
RDH10	8.735334	9.466311	9.813191	31.00519	31.44406	28.9671
SDR16C6	1.025291	0.756863	0.938725	0.214176	0.164624	0.18701
LOC100859726	0.150114	0.025856	0.074916	0.823822	0.585164	0.303467
RDH16	0.392138	0.385	0.665382	0.625436	1.694755	1.010736
ADGRA2	0.671403	0.850823	0.877565	2.315231	2.450586	2.379292
ETS1	5.939837	5.885198	5.904194	26.6992	26.35556	27.45135
FGF1	1.760813	2.003394	1.838123	0.565168	0.887073	1.077679
SASH1	7.923512	7.735945	7.586404	21.84675	20.49091	21.40191
TEK	0.298747	0.185248	0.212043	2.001573	2.167083	1.959445
WNT7A	1.679133	1.685036	2.088898	0.375612	0.346452	0.31977
NR0B1	5.380905	5.787069	5.851106	0.652725	0.600569	0.773833
SALL1	5.126965	4.196119	4.94847	13.42355	12.7975	12.53052
ITGA2B	3.597	3.332356	2.893828	1.449845	1.735903	2.060968
KITLG	2.643048	2.311281	1.866949	5.795003	6.409177	4.459437
ACVRL1	1.820687	2.138214	2.659815	5.109473	6.02566	6.390939
AGTR2	0.319045	0.209825	0.238834	2.141381	2.580756	2.360013

RGCC	14.46799	14.73966	11.46366	174.0632	164.8011	169.0426
ADAMTS9	2.063427	1.985829	2.272019	10.48775	10.72241	10.97636
APOH	1.59562	1.965279	1.592621	0.629763	0.64459	0.574432
DLL4	0.686153	0.616934	0.520496	5.761336	6.440232	6.824559
FGF2	6.909008	9.211483	7.422436	40.81074	36.36245	42.914
FGFBP1	1.00832	0.727026	0.631941	0.217162	0	0.260724
GATA2	23.1876	21.72088	25.32948	9.94272	10.72572	9.570928
RHOJ	1.557167	1.62856	1.244012	5.337845	5.297249	5.742913
