# Supplementary material for: Comparison of primordial germ cell differences at different developmental time points in chickens
Source: Anim Biosci. 2024 Aug 5;37(11):1873–86. doi: 10.5713/ab.24.0283 (PMC11541041; doi:10.5713/ab.24.0283)
Supplement: Supplementary file 6 [file ab-24-0283-Supplementary-Table-6.pdf]

Table S6. Genes related to germline transmission ability during the development of female PGCs from E4.5 to E5.5

gene_id	Expression_ Female5.5-1	Expression_ Female5.5-2	Expression_ Female5.5-3	Expression_ Female4.5-1	Expression_ Female4.5-3	Expression_ Female4.5-2
ACAP3	2.888593	2.589929	2.942941	9.007508	9.435483	8.506845
ADGRL3	0.826182	0.708865	0.598571	2.396714	2.481308	2.273971
ASCL1	1.161096	1.392173	0.793121	2.630361	2.108821	2.736798
AUTS2	4.363239	4.919582	4.873035	12.94383	13.46384	12.59417
BARHL2	0	0	0	0.083076	0.257571	0.248761
CCK	2.676549	2.292239	2.211513	14.99738	15.78698	17.65438
CDK5R2	0.901265	0.71491	0.884976	5.737052	5.867156	6.204703
CELSR1	2.718534	2.817722	2.986702	6.760113	7.352101	7.187515
CHL1	2.703275	2.645502	2.683911	7.48369	7.381289	7.00573
DCDC2	2.149541	2.008159	2.492943	10.10705	10.4564	10.10406
DCLK1	1.190279	1.010621	1.403991	4.250427	4.480646	4.627041
DCX	1.129884	1.204841	1.167223	3.213523	3.54185	3.58756
DRGX	0.634097	0.881115	0.415091	1.579657	1.509899	2.124458
DYX1C1	6.632248	7.847344	7.278545	2.402357	1.770925	2.49845
ESX1	0.370215	0.093723	0.440055	1.34752	1.564317	1.050196
FEZF1	0.178646	0.300752	0.328908	0	0	0.126692
GATA3	0.010398	0.020005	0.006806	0.571314	0.420771	0.412932
GPM6A	0.089261	0.057246	0	1.21205	1.980886	2.447678
KIRREL3	3.897413	3.568825	3.642744	1.292685	1.444499	1.637011
LOC777082	4.705264	3.582298	3.599806	0.054694	0	0.081887
LRP12	13.0479	12.28148	13.18234	24.89206	26.85621	24.12086
MAP1B	6.095232	5.87119	5.63225	27.86046	27.26513	27.00457
MAPK8	25.22187	24.43768	25.21058	6.593476	6.201659	6.293087
MAPT	7.758366	7.669665	7.01252	20.36451	21.91696	21.22866
MDGA1	0.677829	0.620835	0.430434	2.572422	2.547256	2.582938
MEF2C	0.110308	0.134686	0.141642	1.635838	1.333366	1.275722
NR2F1	0.451472	0.169489	0.324375	1.710814	2.759925	1.853369
NR4A2	0.76898	1.103768	0.719125	3.295578	3.178826	3.624092
NTRK2	1.674773	1.720416	1.807042	0.090707	0.061966	0.161124
PAFAH1B1	23.44371	24.10306	23.94755	59.52202	58.71011	59.52956
PAX6	0.995867	1.486855	1.181265	0.166045	0.179403	0.105467
PCM1	44.52189	43.69976	43.80507	17.7615	17.89042	17.29182
PCNT	18.83806	18.58461	18.24701	5.48606	5.707165	5.658743
PEX13	18.56983	20.93906	18.05049	7.917229	8.747047	8.79431
PHOX2B	0.025063	0.024111	0.689082	8.025329	7.705028	7.465168
PRKACA	11.36352	10.56037	9.487977	1.074242	0.578721	0.866904
RAPGEF2	3.682222	3.796332	3.784932	8.397795	9.511938	9.1727
SDCCAG8	4.89438	4.97586	4.447611	1.733965	1.684256	1.840244
SPOCK1	79.1214	82.91839	80.85537	10.77373	11.00096	11.50958
TBX20	0.387045	0.581777	0.427556	1.122922	1.397346	1.646906

TUBB2A	24.33324	24.92359	24.36179	56.76945	55.40969	57.42006
ADGRA2	0.333264	0.271902	0.260964	0.671403	0.850823	0.877565
ATOH8	1.038432	1.316828	1.16533	4.40138	4.65433	4.004167
BCAR1	14.38035	16.60235	14.72597	53.68174	53.46602	55.0467
BMPR2	6.870671	6.544573	6.613758	29.76359	29.58306	28.85179
FGF1	0.017052	0.278868	0.368363	1.760813	2.003394	1.838123
NRP2	13.92634	13.99999	14.06193	57.15878	55.51349	54.62192
PRKCA	15.49621	14.86645	15.725	4.491682	4.236889	4.453985
PTK2B	0.24095	0.221493	0.162988	4.489219	4.634237	4.004856
RHOB	13.33647	13.75365	11.45177	62.49587	63.60705	65.24562
SMOC2	0.609795	0.607207	0.577764	2.46279	1.916773	2.700942
WNT7A	0.154126	0.148269	0.025223	1.679133	1.685036	2.088898
ZC3H12A	7.471867	7.72167	7.060898	24.88739	24.28836	24.90193
ADNP2	19.79641	19.4995	19.17809	8.906639	8.467533	8.384712
AQP1	0.628888	1.020923	0.231571	1.563922	2.039577	1.560982
BRINP1	7.73929	8.192099	7.187982	1.856312	2.280416	2.656878
BRINP3	0.179215	0.218822	0.209817	0.055505	0.060737	0.02933
GJB3	0	0	0	0.307423	0.234104	0.048449
HAND2	0.831597	0.765874	0.754647	12.44078	12.72599	12.01491
IREB2	27.33851	27.7613	28.70206	58.65173	58.76266	59.6352
KLF4	1.598962	2.364701	2.366812	11.10088	10.17565	10.48201
LOC420039	0	0.117077	0	0.658826	0.340439	0.904183
LOC772080	0.406477	0.255675	0.153512	38.03386	34.27109	32.43359
LYN	4.398876	4.459879	4.080949	13.66273	13.16912	14.38992
PCK1	0.011561	0.05561	0	0.361437	0.316938	0.218641
RET	1.897384	1.515916	2.005192	5.839319	5.65694	5.661095
RIPPLY3	0.023139	0.06678	0.059075	0.004384	0	0.008752
SETX	23.85684	23.82819	23.73164	10.58804	9.955594	9.566946
SOX9	6.605411	8.210128	7.193944	22.96322	22.32013	23.84128
STRA8	7.193419	6.068376	5.691478	2.725886	1.85551	1.857443
TBX1	4.481022	4.545379	4.536216	1.544914	1.432748	1.596274
WNT3	0.059452	0.400349	0.408642	0	0	0
WNT7B	0.082786	0.199101	0	1.176414	1.337369	1.330763
YES1	70.62228	69.68557	70.91916	19.74917	20.28819	23.97092
ARHGEF39	15.19172	17.0988	18.73189	2.138048	1.802867	1.550091
ATP8A1	58.84967	57.41125	59.11409	9.112022	10.1341	9.263454
ATVR1	20.28038	20.4436	22.44709	56.39538	54.3187	57.08323
CASS4	0.047859	0.085175	0.136282	0.249366	0.248341	0.248898
CCLI7	0.271137	0.596191	0.494439	1.834731	2.313293	2.527169
CCLI8	0.568457	0.664039	0.877144	6.385423	7.274969	7.256485
CD274	0.461831	0.588808	0.458948	1.692086	1.737827	1.436359
COL18A1	15.7413	15.06426	15.021	56.18157	54.85973	52.23053
CSF1R	2.802597	3.46046	3.03565	13.21367	13.17506	12.41704
DAPK3	19.57846	18.95702	20.27054	41.92161	41.04632	40.01451

EDN1	0.304337	0.739636	0.80214	31.89673	31.52158	30.07984
EGFR	5.974967	5.48605	5.471484	14.24971	14.22354	13.8158
FAM110C	1.068705	0.990613	0.983803	8.183903	7.629485	7.842201
FAM65A	0.88955	1.245224	1.271015	9.923557	9.887243	9.079944
GRB7	0.04461	0.085829	0.146011	10.36807	10.94804	11.20627
GTSE1	31.71625	31.58865	30.6487	6.367	4.64551	4.391399
HOMER3	2.590897	2.60032	2.657076	7.394242	7.061023	6.869718
IKBKAP	54.94615	59.04389	55.75483	13.23618	13.6716	14.03471
IL1B	1.84125	1.444996	2.616802	12.09545	12.09707	13.19523
ITGA5	9.809359	11.39399	10.52588	88.40464	84.10825	88.49162
LEF1	12.98944	13.23396	11.97486	4.84573	4.151305	4.887533
LGR6	0.889931	0.862701	0.826794	2.049333	2.499958	2.420917
LOC100857467	3.476184	4.417885	2.880999	9.154428	8.086988	9.227689
LOC107049953	7.78385	8.974257	8.424991	18.18185	19.93064	17.72067
LOC107050168	0.090782	0.218332	0.022285	1.182541	0.244425	0.278984
LOC107051471	3.523699	3.402742	3.57887	7.555524	8.150776	9.105542
LOC121106433	10.56909	7.58981	8.380417	91.05422	105.6157	97.26412
MCAM	3.297832	3.539708	3.418131	40.55693	40.62889	40.56732
MIEN1	12.95559	16.07841	15.71472	31.26888	32.36695	31.65739
MYO1F	1.0241	0.960138	0.988547	6.215761	6.13491	5.687071
MYOC	4.920394	4.96879	5.458757	2.704101	2.68815	1.915018
NEDD9	1.691605	2.038764	1.692373	3.991254	4.493583	4.587351
NTRK3	10.15702	10.08656	10.17796	0.649339	0.631509	0.7701
ONECUT1	1.149255	1.046881	0.958712	0.125255	0.278809	0.221188
PAK1	8.617931	8.868866	9.205624	19.17645	19.69477	19.30184
PDCD1LG2	0.859865	0.759665	0.964942	2.70978	2.766118	2.505568
PDGFA	3.183136	2.903107	3.328568	38.9281	40.15013	34.43782
PDGFB	0.484466	0.521984	0.751622	31.76009	31.84604	31.82866
PDPN	2.837736	2.509479	2.544149	12.99087	14.71994	14.13309
PIK3R1	0.83936	0.803877	0.783897	2.915629	3.685258	3.255839
PIK3R3	6.73552	7.306433	6.510251	23.43203	22.73977	23.40612
PODXL	3.283171	3.259342	3.478347	23.08189	22.97836	22.00563
RHOC	7.451008	7.798855	7.11025	30.62103	30.15613	31.01001
RHOD	0.035986	0	0	0.204548	0.140929	0.170136
RLTPR	0.27941	0.268793	0.358348	0.632039	0.658777	0.496054
SEMA3C	0.322031	0.548097	0.700529	10.15204	9.953422	9.940881
SEMA3D	1.064151	1.201752	1.41594	11.87107	13.03094	12.08938
SEMA3E	0.0246	0.04733	0.012078	0.559318	0.252892	0.325655
SEMA5B	1.07102	1.372857	1.115484	2.232732	2.664374	2.837775
SEMA6B	5.757079	5.976159	5.621667	1.981894	2.173314	1.645958
SEMA6C	1.37866	1.648368	1.67284	0.466457	0.539918	0.819421
SEMA6D	4.153451	4.542774	4.181263	1.985119	1.919677	1.680641
SPHK1	14.03212	14.04813	13.05566	39.05467	38.74387	37.07765
TGFB3	24.47428	24.93587	21.79375	115.237	121.0826	117.1066

TGFBR2	0.860505	1.150512	1.195824	13.70647	15.06485	13.92205
TNFAIP6	3.930835	4.092884	4.086839	20.59094	18.52957	18.21278
TWIST2	0.13358	0.185618	0.262332	0.534313	0.319693	0.435068
VIL1	0.384109	0.250315	0.121667	1.044708	1.067545	1.007596
VILL	0.499408	0.266185	0.490382	25.22222	24.3485	24.64515
VSIR	0.812955	0.360952	0.807034	5.865035	5.361623	5.566801
WNT11	0.076567	0.099654	0.106141	0.452283	0.493879	0.315151
WNT5B	2.734219	4.126241	4.504361	46.99322	45.369	44.54004
ZNF703	2.777621	2.095985	1.751554	6.771606	7.098057	6.011907
ADD2	5.05973	4.490774	5.277388	29.85846	31.9704	32.7862
CD244	0.299472	0.864276	0.709201	2.019317	2.224883	1.549119
CD74	9.066306	10.50483	9.538337	23.48225	24.96785	23.12138
DBH	0.503743	0.149108	0.215612	14.10867	14.36592	13.78902
F11R	46.36485	51.03113	44.23249	147.016	141.7009	153.5307
GRB14	0.271466	0.25742	0.159936	0.091848	0.037969	0.168682
INPP5D	0.780044	0.782201	0.804893	2.880763	2.731244	3.087874
ITGA4	21.00875	20.68196	20.43543	4.307186	5.019494	5.039578
JAM2	11.86958	9.268306	9.233228	2.833079	3.21466	3.221308
JAM3	9.582147	9.25385	10.19122	46.88374	48.4603	49.18222
JAML	0.029174	0.028066	0.019098	0.902848	0.914026	1.177014
LOC107049116	0.017443	0	0	0.198295	0.290321	0.065974
LOC107049666	4.062638	3.954929	4.227375	0.643374	0.344328	0.401353
LOC121107557	0.512548	0.417214	0.154857	1.456693	0.984333	1.155707
PDE4C	0.245239	0.378902	0.408642	3.449721	3.317807	3.801614
PPIL2	24.50354	24.00411	22.421	9.888921	9.600527	9.689481
SELPLG	0.108692	0.261404	0.177879	0.875242	0.940009	0.650915
SLC7A10	1.815647	2.043586	2.406823	0.688024	0.248869	0.343365
SLC7A6	9.316074	10.56284	8.691984	19.46355	18.90701	19.40556
TNFRSF10B	6.518791	6.757415	6.505481	37.98626	35.06363	33.41137
TPPNR1L	2.655977	2.937306	3.008412	7.578171	7.330177	6.377428
VPREB3	0.729945	1.170347	0.637114	0.268923	0	0
VSIG10L	3.295234	3.432506	2.607091	16.51348	16.12094	15.7382
ANXA6	13.82297	13.5644	11.74194	67.0639	64.53424	64.0187
CORO1C	14.66737	14.638	14.67173	43.55083	42.81218	46.51135
FOXS1	0.222376	0.556207	0.764248	1.853883	1.023283	1.871425
GDNF	0.155265	0.280059	0.104815	2.022494	1.824166	1.871879
ISL1	0.16723	0.160875	0.135649	0.799016	0.918302	0.955642
KITLG	0.45068	0.846465	0.758631	2.643048	2.311281	1.866949
LAMA5	9.223996	9.736983	9.737777	26.97103	27.17275	27.00013
OVOL3	0.980921	0.689588	0.765614	2.501906	2.8319	2.758815
SNAI1	27.82067	29.40578	30.47884	9.177046	7.864958	9.830025
SOX10	0.007225	0.013901	0	3.134764	2.595995	3.046893
ABI2	36.19153	36.63678	35.74886	12.29439	13.35051	12.13032
ADAMTS12	3.04199	2.974176	3.015459	6.363577	6.819701	6.742966

ADGRG1	11.07709	11.40378	10.84272	38.37152	36.52155	36.80778
ASAP3	2.335034	2.468164	2.561745	8.240772	9.201035	7.809589
BAMBI	6.243625	6.693452	6.696352	51.51004	54.70034	53.19961
CD63	115.5992	122.7778	121.5653	639.1738	636.6078	641.8509
CDH7	3.614559	4.002656	3.699088	0.461642	0.519036	0.420267
CLEC14A	3.201816	2.456068	2.917913	8.842033	6.822758	7.974533
COL5A1	11.35894	11.21363	10.57667	41.31956	42.92299	42.1583
DGKZ	42.72049	42.53066	44.21828	17.59487	18.18935	17.8136
ELMO1	9.12327	9.273908	9.263889	2.534847	2.301018	2.041265
ELMO3	1.779082	1.948957	1.412588	4.967556	5.558868	5.425064
EMP2	2.896833	2.401047	2.647628	7.435322	8.017861	6.862139
ENPEP	0.073705	0.023635	0.016083	0.612909	0.424955	0.456882
EPHB3	6.195728	6.226759	6.319944	26.39916	24.66368	25.6603
FN1	58.86355	61.20262	59.23008	599.5264	600.0516	596.8475
GOLPH3	49.31352	50.15384	49.86023	20.05264	20.86852	21.38061
HOXA5	0.039427	0.104304	0.087107	2.493185	2.306427	2.721505
ITGB4	0.366316	0.578626	0.377458	27.75388	27.83714	27.75302
ITGB6	0.185	0.164948	0.221532	37.74087	37.10449	36.25342
ITGB8	0.811866	1.004164	1.006845	2.317364	2.36396	2.245711
JUP	33.58089	34.45852	35.64056	151.393	151.724	150.3813
LIMA1	14.11543	12.0645	13.16244	55.54322	54.77752	58.49276
LOC100858919	0.222608	0.149904	0.295089	1.702926	1.759925	1.852331
LOC107049174	0.947239	0.87551	0.948357	5.313841	5.027999	5.321416
LOC107049717	1.95913	2.70529	2.448374	5.123953	5.451477	5.850004
LOC121108098	1.670055	1.762459	1.713299	3.624738	5.161514	4.195379
LOC418421	0.039835	0.011496	0.011734	0.403792	0.401707	0.387966
MYO18A	7.658798	7.012003	7.613275	20.94793	21.79223	21.734
NANOS1	9.888834	8.551076	8.619087	43.19911	47.01654	48.62322
NOX1	0.024227	0.046613	0.015859	1.729015	1.288773	1.412684
NR6A1	130.0195	128.8434	131.2467	8.821726	9.125016	8.499383
PALLD	4.999168	4.552995	5.659072	44.89463	44.72592	45.00015
PARP9	2.252194	3.246274	2.755055	5.768692	5.056003	5.643122
PIK3C2G	0.054574	0.090682	0.155891	0.427705	0.398305	0.417519
PIK3CG	1.230737	1.216729	1.261037	4.728293	4.224537	4.521614
PPP1R19B	6.072066	5.893646	6.095808	12.19165	11.88987	11.82595
PRAG1	0.48177	0.774395	0.592826	4.223207	4.263062	4.63622
PRKCZ	12.00461	12.24388	12.55337	6.071868	5.299841	5.403816
PSTPIP2	0.045891	0.220736	0.225308	1.550607	1.602508	1.446441
PTPRF	43.65683	43.87785	44.47845	20.07653	20.69895	20.00049
RASGEF1A	3.997156	3.576995	3.597391	1.673203	1.391932	1.271933
RHBDF1	1.253226	1.545136	1.530807	5.59652	5.593512	5.600278
RPS6KB1	9.975368	10.22145	9.772692	25.26175	24.95764	23.99613
SH3BP1	0.120794	0.063384	0.075479	0.114434	0.376296	0.238822
SHROOM2	6.711303	5.786902	6.703882	16.72425	16.74563	17.11023

SIX2	0.101985	0.065407	0.200284	0.161027	0.49925	0.739332
ST14	3.753233	3.697852	3.334174	38.10024	38.07335	38.49071
TGFB2	2.807269	2.673725	3.078813	29.84966	30.75305	28.8427
TNFAIP3	0.565267	0.652546	0.421839	9.939038	9.645541	9.398071
ZRANB1	8.229168	9.106298	8.717737	16.83319	17.72033	16.88839
ABHD2	6.640342	7.100392	7.283219	27.42478	25.97357	25.75471
ACVRL1	0.436788	0.06723	0.171557	1.820687	2.138214	2.659815
ARAP3	0.28961	0.437808	0.482424	3.052293	2.987367	2.621113
BCL2	1.398558	1.400896	1.604522	3.983896	4.084305	4.003675
DLG5	9.418738	9.715326	9.612898	4.305192	4.273397	4.049244
DPEP1	0.019032	0.036618	0	0.270456	0.167705	0.143972
DPYSL3	327.3633	331.1186	326.9927	100.7183	96.83356	92.90903
FUZ	5.867563	5.881203	5.623516	2.16355	2.04677	2.192706
GTPBP4	85.46389	81.62253	78.39946	36.19578	34.35968	35.12178
HNF4A	0.022352	0	0.007316	0.268215	0.692983	0.246576
KANK1	24.42374	25.29427	23.53338	5.638493	6.458703	5.959855
LDLRAD4	7.882741	8.424679	7.577642	19.78487	20.05184	19.06496
LOC107050437	0.66655	0.944779	0.852943	0.295576	0.312412	0.318488
LOC112530680	0.015672	0.04523	0.076944	0.193011	0.138096	0.414935
MCTP1	0.048436	0.151434	0.142681	0.521949	0.557203	0.629742
MIA3	8.142718	8.24997	7.377754	16.32214	17.10504	16.39924
MITF	4.127763	3.959759	4.235325	8.298729	8.44595	8.337949
NAV3	1.391112	1.34083	1.405449	5.317211	5.298366	4.959992
OSBPL8	41.04793	40.16263	40.30606	17.05476	16.83192	17.6822
PTPRT	0.193891	0.209209	0.339609	2.2886	1.926537	2.098482
SCAI	13.08438	12.85903	12.46589	5.571205	6.379158	5.630436
SRGAP3	11.45472	11.62258	11.1181	32.17545	33.28933	32.17292
STC1	0.395665	0.546724	0.381451	2.71926	2.831407	2.646125
SULF1	1.20883	1.470166	1.785299	4.548175	4.868795	4.77195
TBX5	0	0.038172	0	0.714224	0.679856	0.775415
THY1	7.420991	8.426119	7.378528	21.09092	23.95979	21.84566
TIE1	0.17712	0.127792	0.09179	0.316947	0.250483	0.288438
TMEFF2	17.17897	17.04114	17.07629	6.498035	7.203049	6.426966
ZMYND8	17.72476	17.67808	17.74781	8.177775	8.41638	8.467646
CAMK2A	0.049543	0.079434	0.016216	0.297255	0.080843	0.327927
FLRT2	0.753685	0.818731	0.765009	3.754549	4.068841	4.02375
IGSF10	0.206831	0.238055	0.290133	0.006998	0.079554	0.020954
KIF20B	24.31831	22.61444	23.92886	3.086229	2.575583	2.984014
LOC768380	3.095269	3.124438	3.108889	0.340727	0.24009	0.252488
SCRT2	0.31214	0.07507	0.65131	1.18283	0.955016	0.811667
SOX14	0.100174	0.192734	0.491816	0	0.065384	0
UNC5D	0.117308	0.170801	0.183678	0.273326	0.34766	0.359752
CCL5	1.736705	0.222762	0.795815	10.74913	13.26269	14.77964
LOC101749460	0.036142	0	0	0.256793	0.271286	0.381618

P2RY2	0.073176	0.059565	0.088435	3.103562	3.108243	3.204179
TMSB4X	644.6062	659.2647	643.9317	2197.679	2212.858	2229.475
BCL2L1	10.9617	11.45658	11.91123	38.0741	37.21592	37.63414
BOLL	16.03163	16.30462	16.28215	2.917947	2.66705	2.540116
DND1	253.8742	267.8744	263.4998	27.88276	25.37797	27.55979
DZIP1	16.93549	17.47467	17.18154	7.448635	6.643565	6.650619
MOV10L1	45.95052	46.12296	45.51745	11.71914	11.67495	11.95779
PRDM14	48.36862	49.92274	48.22401	0.703884	0.64592	0.857003
TDRD1	20.83687	20.27069	20.1555	5.887336	5.956087	5.667568
EFNB2	76.31659	74.06094	73.65337	12.38718	11.64651	10.19268
EGR3	6.399873	7.320321	7.061634	1.750129	1.937901	1.767635
GREM1	4.700289	4.941385	4.049536	0.14037	0.096712	0.264644
MMRN2	0.088839	0.059824	0.043617	0.277734	0.191354	0.23101
GNA12	44.17673	43.7788	44.1804	15.97921	15.42593	16.40592
PRR5L	2.123045	2.563829	2.19873	6.455644	6.020978	6.187234
RFFL	3.85994	3.973987	3.991784	10.86127	11.61076	10.38268
SDC4	4.830594	4.807649	4.754218	78.89343	79.62706	79.19769
WDPCP	1.210923	0.966218	1.203758	0.325059	0.318	0.313423
CYP26C1	2.033524	2.068039	1.321673	0.605461	0.701572	0.567697
DHRS3	0.763874	0.596398	0.641362	17.44123	18.73495	18.67508
EZH2	37.57101	38.38882	37.42901	15.22905	14.68699	15.44053
ZNF536	0.06446	0.058747	0.063295	0.359973	0.315554	0.221352
ADAM8	2.408741	2.232642	2.261621	27.05837	27.18942	26.02663
MYH9	62.88916	62.90585	61.25304	282.8884	282.8489	281.498
VHL	23.69952	23.18774	21.62718	51.52163	54.094	52.35284
LOC121109863	11.79219	11.4258	11.22169	1.781444	1.532247	1.835454
MACF1	11.74153	11.31468	11.82384	27.86315	27.29061	27.34916
PTK2	96.76657	95.91922	101.6096	35.78932	36.54644	35.7315
CENPV	3.457255	2.237978	2.950596	8.387076	7.623871	8.157446
SGPL1	15.24499	13.8957	14.43139	30.52971	30.56898	30.4141
WASF2	25.3381	24.92742	25.62606	52.77396	52.08881	51.10516
DRD4	1.315661	1.216988	1.267039	0.431445	0.222943	0.191393
NFE2L2	13.14817	12.96293	13.37583	57.20251	59.29847	59.85549
MYT1L	1.70495	1.775032	1.620848	0.814022	0.72172	0.889465
