# Supplementary material for: Comparison of primordial germ cell differences at different developmental time points in chickens
Source: Anim Biosci. 2024 Aug 5;37(11):1873–86. doi: 10.5713/ab.24.0283 (PMC11541041; doi:10.5713/ab.24.0283)
Supplement: Supplementary file 7 [file ab-24-0283-Supplementary-Table-7.pdf]

Table S7. GO terms related to cell proliferation during the development of male PGCs

	Term_description	ListHit	Enrichment_score	p-value
E3.5-4.5	chondrocyte proliferation	5	4.522453	0.001553
	activated T cell proliferation	4	5.065147	0.002713
	positive regulation of activated T cell proliferation	7	2.954669	0.004836
	positive regulation of neuroblast proliferation	6	3.165717	0.006018
	positive regulation of B cell proliferation	9	2.374288	0.00823
	positive regulation of cardiac muscle cell proliferation	4	3.165717	0.025476
	negative regulation of cell proliferation	40	1.266287	0.064415
	regulation of epithelial cell proliferation	3	2.713472	0.083845
	neural precursor cell proliferation	3	2.713472	0.083845
	positive regulation of blood vessel endothelial cell proliferation involved in sprouting angiogenesis	3	2.713472	0.083845
	positive regulation of vascular endothelial cell proliferation	3	2.713472	0.083845
	positive regulation of endothelial cell proliferation	8	1.688382	0.088841
	meiotic cell cycle	13	1.469797	0.093945
	negative regulation of fibroblast proliferation	5	1.978573	0.094429
E4.5-5.5	positive regulation of cell proliferation	107	1.327668	0.000209
	negative regulation of neural precursor cell proliferation	7	2.540561	0.003205
	positive regulation of endothelial cell proliferation	18	1.742099	0.003664
	G2/M transition of mitotic cell cycle	29	1.530935	0.003975
	cell proliferation	70	1.294553	0.005152
	negative regulation of B cell proliferation	6	2.488713	0.008221
	regulation of epithelial cell proliferation	6	2.488713	0.008221
	G1/S transition of mitotic cell cycle	20	1.569458	0.011071
	positive regulation of cell cycle	16	1.659142	0.01145

	negative regulation of epithelial cell proliferation	17	1.592241	0.015602
	negative regulation of fibroblast proliferation	10	1.814686	0.020258
	positive regulation of cardiac muscle cell proliferation	6	2.177624	0.023267
	positive regulation of smooth muscle cell proliferation	15	1.555445	0.029003
	regulation of cell proliferation	36	1.274707	0.047108
