# Supplementary material for: Comparison of primordial germ cell differences at different developmental time points in chickens
Source: Anim Biosci. 2024 Aug 5;37(11):1873–86. doi: 10.5713/ab.24.0283 (PMC11541041; doi:10.5713/ab.24.0283)
Supplement: Supplementary file 8 [file ab-24-0283-Supplementary-Table-8.pdf]

Table S8. Genes related to cell proliferation during the development of male PGCs from E3.5 to E4.5

gene_id	Expression_ Male3.5-2	Expression_ Male3.5-1	Expression_ Male3.5-3	Expression_ Male4.5-1	Expression_ Male4.5-2	Expression_ Male4.5-3
ACVRL1	5.863852	6.198249	5.622993	1.725056	1.982807	1.204948
ADORA2A	3.119554	3.039691	3.282527	1.011038	0.982951	1.005597
ALDH1A2	57.85343	57.96187	58.72447	22.19439	21.9233	23.59711
BMP7	3.721651	3.784161	3.895853	6.311145	6.683954	6.269245
CCL26	30.67341	29.24594	33.89501	5.029186	5.999797	5.460544
CDH5	32.71398	32.45791	32.26043	0.286649	0.284155	0.258127
CGREF1	1.568005	2.43929	1.723371	0.634397	0.690198	0.944966
CLMN	5.243834	5.801098	6.043576	1.943778	2.488936	2.101026
CTBP2	21.25643	21.55991	21.15383	5.124083	4.974792	5.064989
DLEC1	1.078319	0.998079	0.757305	0.387499	0.257962	0.3626
DLL1	2.448039	2.851187	2.5516	5.456768	5.173186	5.754345
DLL4	6.702903	6.313609	6.947604	1.166416	1.059915	0.970068
E2F7	5.366569	5.360629	6.307348	1.926956	1.669444	2.287657
FEZF2	1.557122	1.896386	1.928842	0.735319	0.223503	0.205702
FGF2	46.03171	47.8621	51.45571	9.179021	8.645736	8.958738
FGFRL1	48.41067	48.32796	50.74353	7.205835	8.029727	7.691061
FTH1	794.6204	773.8091	774.0829	1675.259	1630.269	1627.579
GDF11	2.594829	2.153609	2.114277	0.773955	0.930667	0.93441
HNF4A	1.87375	1.901716	2.250923	0.428524	0.437608	0.834055
ING5	6.513032	8.076986	6.995942	11.94293	13.16529	12.07845
ITGA1	8.629787	9.330225	8.953257	1.872582	1.75022	2.155899
LOC100858919	4.79288	4.758903	4.864388	1.701795	1.833809	1.613124
LOC112530287	2.396688	3.232996	3.539672	1.117825	1.038672	1.185987
LOC770492	0.93963	1.181417	1.032998	0.202245	0.210033	0.178435
MAGI2	10.18906	11.10319	10.50332	1.528338	1.633997	1.226155
MYOG	0.134457	0.164348	0.45303	0.76937	0.769403	1.099325
NPM1	993.8782	983.8548	970.204	407.5192	402.8747	418.3264
PAX6	0.295498	0.205555	0.335685	0.030548	0.087243	0.023889
PTPRVP	2.712222	2.873622	2.938403	5.385086	5.404036	5.550457
RERG	14.44905	13.65711	14.04051	4.061594	4.375256	4.466165
SERPINE2	457.7954	447.7083	445.4767	181.5001	184.8682	183.6849
SFRP4	1.742965	1.547104	1.850614	0.422151	0.863113	0.839074
SFRP5	1.215886	0.227478	0.713114	0.157763	0.054613	0.027416
SPRY1	28.11776	29.80962	28.1428	7.611763	8.64935	8.390194
TBX5	0.017948	0.048751	0.100788	0.564213	0.717611	0.449485
TGFB3	168.5724	169.1479	167.5231	64.43011	65.36925	67.74986
TNS2	19.08101	19.7689	20.71283	5.952655	6.620083	6.086275
TP73	0.971228	1.228521	0.817781	2.058298	2.067702	2.2724
TSPAN32	0.34495	0.77147	0.490463	4.433938	5.042037	4.996038
ZNF503	22.14275	23.13521	21.02349	8.182955	8.381062	7.037231

HORMAD1	3.439302	2.993956	3.663517	8.862179	10.55394	8.72152
HORMAD2	0.263884	0.336725	0.465533	0.47935	0.714802	0.602336
KIAA0430	11.13169	11.73972	12.19817	19.06546	20.1121	20.05914
KLHDC3	7.901093	7.891047	8.22239	14.22589	14.51939	14.52917
PDIK1L	3.500374	3.404384	3.459576	6.721763	7.332016	6.789444
PLD6	6.238987	6.416666	6.662908	11.50517	10.04849	11.46087
RAD21L1	0.197884	0.289199	0.093786	0.738458	0.454455	0.446778
REC8	1.458003	1.250054	1.070643	3.145481	2.527867	2.816177
RSPH1	0.952766	1.065158	1.611998	0.492481	0.639306	0.077025
SMC1B	0.514259	0.376679	0.824553	2.002152	2.079249	2.381284
STRA8	0.525606	0.701088	0.458856	2.028894	2.286048	2.322898
SYCP3	0.881473	0.673395	0.635288	2.504929	2.38093	2.65612
TDRD1	2.33363	2.55497	2.572278	5.95059	7.106689	5.818526
ACVRL1	5.863852	6.198249	5.622993	1.725056	1.982807	1.204948
EGFL7	9.426144	10.2092	8.519785	0.901639	1.057964	1.074429
NRARP	13.00556	13.92265	14.03125	4.494279	5.496868	4.816565
NRP2	100.0336	103.2791	103.404	40.42377	41.96202	43.26562
POLD4	7.617112	6.216316	7.13914	11.86394	12.37128	11.66058
VEGFA	118.4564	119.0424	122.6594	31.44819	29.68081	27.37701
VEGFC	8.668518	9.288697	9.509763	3.609585	2.698394	3.456123
APLN	3.599282	2.452733	3.190033	0.993771	1.199395	1.0922
GHSR	0.153224	0.277463	0.523011	0.072161	0.112409	0.056431
ITGA4	8.700017	8.321839	8.414447	3.05135	3.454886	3.260432
APELA	6.434671	6.241151	7.647739	1.920838	1.633173	2.729
APLNR	3.026431	2.742394	2.960177	0.749669	0.692033	1.281068
GATA2	8.628703	8.317926	8.984816	16.4174	16.98172	16.17095
SLC39A5	0.366248	0.540288	0.63828	0.080294	0.264057	0.15349
HES5	0.319837	0.225542	0.341332	0	0.027074	0.027183
LOC107057363	0.533725	0.73226	0.71241	0	0	0
LOC419390	3.642412	2.750921	3.046108	0.640134	1.877037	1.315286
AGTR2	2.741962	3.237265	2.98483	0.212775	0.182871	0.359562
MORC4	16.91284	16.59917	16.13893	33.31724	32.82684	33.19362
NLRC3	0.390033	0.280435	0.284201	0.893442	0.94047	0.659076
TREMB2	5.709459	5.311668	5.445941	1.771063	2.324827	2.053487
MEF2C	4.885858	5.113514	4.663016	1.21602	1.16148	1.340015
TBX20	0.328255	0.267486	0.325293	0.463775	1.035511	1.039677
ZFPM2	1.564047	1.75544	1.818982	0.400259	0.584538	0.313008
BST1	10.35867	10.94996	10.79912	3.759907	4.460517	4.125021
CARD11	5.214749	5.135367	6.073657	1.008849	1.136989	1.231214
CD74	62.98821	59.50873	59.83745	17.5103	16.45058	17.1194
GPR183	4.593158	4.563442	3.958876	0.746614	0.702217	0.690353
IL7	0.060988	0.046591	0.015109	0.355438	0.167784	0.08423
LOC101748032	0.901555	0.894463	0.905024	0.210915	0.25769	0.206982
SLC39A10	22.9985	22.72112	20.84247	6.41358	6.189112	6.67116

TFRC	91.82145	94.82216	90.78935	223.9873	225.6758	225.7742
DCT	0.557506	0.283935	0.357484	0.086876	0.252618	0.108701
DISC1	4.818053	4.239915	4.782704	1.580238	2.128005	1.412306
OTP	0.138034	0.261115	0.019541	0.522379	0.347196	0.283232
SOX10	6.830023	6.184731	6.930325	1.966895	2.359099	2.505794
IGF2	8.567137	7.807153	8.202828	1.77469	1.769123	1.929284
IL23A	0.340957	0.07442	0.289613	0.95484	0.723606	1.453034
IL6R	13.43736	13.43221	12.78906	4.4755	4.492911	4.33815
SLAMF1	0.200324	0.031392	0.198517	0.016329	0	0.017026
SLAMF8	3.036282	2.518627	2.053972	0.635765	0.840314	0.863782
STAT5A	15.99254	15.09017	15.27124	39.83112	41.64159	40.61017
TMIGD2	0.621684	0.664903	1.108936	0.263504	0.273651	0.274751
BG8	2.673415	3.543328	3.527074	7.133319	7.354834	7.33104
BTN3A3L2	0	0.018221	0.141819	0.60658	0.669309	0.968473
LOC121106920	0	0.010513	0.010228	0.065617	0.045429	0.136836
MOGL	0.094049	0.17962	0.05825	0.772348	0.905584	0.896238
DDR2	6.536544	6.690027	6.850828	2.378079	2.409205	2.470916
IHH	0.029895	0.28928	0.103688	0.538521	0.39477	0.462418
MMP16	2.893206	2.753518	2.648625	1.11057	1.007768	1.247914
SDC3	3.297697	3.708579	3.169364	1.217492	1.313636	1.143064
STC1	6.442871	6.88083	6.965174	2.186052	2.255907	2.602932
