# Supplementary material for: Comparison of primordial germ cell differences at different developmental time points in chickens
Source: Anim Biosci. 2024 Aug 5;37(11):1873–86. doi: 10.5713/ab.24.0283 (PMC11541041; doi:10.5713/ab.24.0283)
Supplement: Supplementary file 9 [file ab-24-0283-Supplementary-Table-9.pdf]

Table S9. Genes related to cell proliferation during the development of male PGCs from E4.5 to E5.5

gene_id	Expression_ Male5.5-2	Expression_ Male5.5-1	Expression_ Male5.5-3	Expression_ Male4.5-1	Expression_ Male4.5-2	Expression_ Male4.5-3
ACKR1	0.056127	0.043378	0.210836	38.16221	39.57087	35.8655
ADCYAP1	2.755507	2.239594	2.457543	14.70837	12.83416	10.9846
ADM	4.206499	4.724821	4.26637	15.63456	13.43168	14.42445
AGTR2	0.070276	0.057934	0.015085	0.212775	0.182871	0.359562
ALDH1A2	12.47412	13.53934	12.58633	22.19439	21.9233	23.59711
AREG	0.302329	0.38572	0.401735	4.884937	4.714026	4.921056
ARNT2	29.57659	30.47798	29.53438	7.843076	7.867219	8.818658
BAMBI	9.963442	8.218034	8.831694	67.73562	63.69255	62.63618
BCL2L1	14.76366	14.56015	13.85589	27.69555	25.27144	26.47051
CCNA2	78.33821	77.28886	77.79268	19.65667	20.44692	20.84707
CCNB1	117.2723	117.6541	118.3545	27.73265	28.53031	28.02489
CCNB3	34.76086	33.89208	35.3407	10.17049	11.71246	12.28456
CCND2	23.35693	24.37312	23.98776	6.660111	6.380674	6.723488
CCNE1	15.964	14.2079	13.44479	5.250858	4.733653	4.348211
CCNE2	8.4128	7.830604	8.297369	1.515542	1.180426	1.376718
CCNF	23.69284	22.57715	23.85037	8.974917	9.379881	9.90297
CCNJL	10.13924	11.29168	9.761591	1.630584	1.584708	1.454704
CDCA7L	137.3521	143.8338	138.0035	20.11637	19.79891	18.79497
CDKN1B	32.58481	34.39078	32.67791	79.37037	74.42544	76.52544
CHRNA7	0.671115	0.745597	0.990389	2.923098	3.092505	3.184852
CNOT6L	3.233054	3.570624	3.131482	11.15175	11.12531	11.0083
CNTF	0.651616	0.308515	0.207916	1.048044	1.126591	0.939407
COX17	43.53364	43.41515	45.45861	73.91583	77.6745	75.60522
CRLF1	3.777166	4.124922	3.345624	7.814458	7.10801	6.900292
CSF1	3.279036	3.27532	3.594833	6.563317	7.317712	5.299584
CSF1R	1.644756	1.858903	1.978794	6.813336	6.788067	6.837033
CSF3	0.141286	0.242653	0.252728	2.728882	2.450995	2.794096
DLL1	2.42523	2.46854	2.443546	5.456768	5.173186	5.754345
DOT1L	11.87214	12.03066	12.3583	22.00796	23.04668	23.82413
DPP4	1.198833	1.171313	1.162762	2.89264	3.081053	3.238453
DRD3	3.463304	3.424948	3.97183	1.239289	1.04475	1.109761
EDN1	1.000071	0.712292	0.505101	16.0455	16.91849	15.01733
EDN2	0.228881	0.168469	0.152068	15.2934	14.38152	14.97329
EFNB2	70.18839	74.98281	70.33366	6.847942	9.207029	9.371572
EIF5A	449.8446	451.7157	451.2977	146.0116	144.8246	142.9129
EREG	0.611928	0.497824	0.345663	1.983871	2.374542	2.068552
ESM1	0.309933	0.135494	0.12096	0.049027	0.010183	0.020448
EZH2	35.7518	34.20788	33.57829	12.13309	11.79334	11.50889
F2	0.098128	0.080895	0.084254	0.901553	0.585168	1.34596
FAM98A	43.49642	43.5173	44.3433	18.34758	17.48518	18.3224

FAM98B	31.98464	34.0811	33.12425	8.517585	9.119761	8.97691
FBXO5	9.625066	8.857544	8.666204	3.03021	2.656033	2.990774
FGF1	0.219191	0.387208	0.134428	0.997103	1.001548	1.295319
FGF18	0.533461	0.507432	0.572543	0.771163	0.934335	1.831516
FGFR2	19.43579	20.01232	18.94441	5.247746	5.471997	5.488444
GDNF	0.285117	0.293806	0.306005	2.52091	1.642277	2.036856
GLI1	2.146993	2.318906	1.80523	0.533413	0.454366	0.343708
GLP1R	5.205864	5.734487	5.587258	0.541428	0.973171	1.541624
GLP2R	0.039858	0.007701	0.016042	0.067621	0.064823	0.02983
GREM1	1.783698	2.507861	1.890041	0.078909	0.204868	0.131643
HBEGF	2.085464	2.223126	1.543621	19.85845	24.44368	23.75918
HCK	0.011937	0.049202	0.051245	0.411256	0.427092	0.558752
HCLS1	0.84295	0.423371	0.661424	1.982004	1.858637	1.912381
HES5	0.224734	0.257315	0.214399	0	0.027074	0.027183
HIPK1	16.16658	17.07361	16.6019	32.8342	32.94698	34.90879
HTR1A	0.351644	0.588837	0.542522	0.091782	0	0.023925
ID4	47.88465	43.16935	43.82525	98.2607	100.0842	90.95546
IL34	0.639894	0.879194	0.406977	7.67045	6.372653	5.546904
ITGB1BP1	5.061867	4.943752	5.248312	9.865813	9.887474	9.33737
KIF20B	21.70098	19.79618	20.52267	3.635999	3.725258	3.730052
KIT	30.88211	32.05111	31.01531	5.491099	5.576382	5.947158
LEF1	10.80179	10.6343	10.33303	2.658069	2.505822	2.74462
LGALS3	3.202961	3.861051	5.091565	78.45784	78.26835	71.83998
LIF	0.490894	1.731578	0.587649	17.03109	17.15467	17.32644
LOC101749460	0.011061	0.005699	0	0.15013	0.155911	0.102352
LOC107054855	0.216934	0	0	0.452973	0.940832	1.121732
LOC107057363	0.232158	0.136705	0.237301	0	0	0
LOC121106810	18.64808	16.26607	32.08812	3.597577	3.269096	3.944213
LOC419390	3.444378	3.270605	2.88383	0.640134	1.877037	1.315286
LOC776507	0.245355	0	0.153609	1.174061	1.795653	1.135144
LYN	3.0538	3.349893	3.494547	9.15884	9.831943	9.651376
MATK	0.379117	0.450121	0.601492	1.936031	2.591417	1.63288
MRGPRH	0.048079	0.136247	0.090303	3.200016	3.622983	3.179591
NOLC1	72.14954	69.97657	72.13617	26.37571	24.51373	27.50156
NOX1	0.059318	0.022922	0.063664	0.820578	1.181791	0.887891
NTRK2	3.745954	3.703502	3.72454	0.076227	0.122782	0.087591
NTRK3	13.35584	13.38642	13.72934	0.870101	0.994895	0.997343
OSMR	4.250929	4.264078	4.20503	16.67863	16.65047	17.42291
OSR2	0.513036	0.382831	0.398726	1.66229	2.014017	1.762132
PDGFA	5.01025	6.649247	3.381189	27.66023	26.17381	21.27946
PDGFB	0.79178	0.504214	0.525149	24.0563	23.42967	22.06154
PGF	4.70783	4.851308	5.42548	43.24246	45.81412	44.33212
PLACL2	33.69062	41.24247	39.01216	7.065114	5.136019	5.051442
POU3F2	7.944076	7.410734	7.818347	3.215502	2.641176	2.415335

PTGFR	0.737266	0.634342	0.614586	1.464731	1.637547	1.706471
PTK2	115.1291	115.0123	116.5539	28.75256	31.85171	31.73985
PTK2B	0.117991	0.162115	0.105529	2.740894	2.841107	2.51537
RAB25	0.164218	0.018803	0.039167	6.324582	5.697649	5.958927
RHOG	2.638467	2.614877	2.723448	6.261937	6.503066	5.069568
ROGDI	6.491675	7.567408	8.009619	12.95025	11.36138	12.59417
RTKN2	6.724803	7.36916	6.596078	2.810004	2.532225	2.495567
RUNX2	0.813187	0.601447	0.608823	1.896547	1.667386	1.881124
S1PR3	3.09822	3.727936	3.628852	1.041092	1.267245	1.307686
SLC35F6	4.956849	4.781879	5.342638	26.42615	24.69937	22.8929
SPDYA	10.06491	10.79427	8.839906	0.981233	0.759631	1.413761
SPHK1	14.49774	15.86353	17.60294	32.2597	32.57464	31.09893
SSBP4	46.15788	47.30494	43.07222	17.9127	18.02039	19.35712
STAT5A	22.79265	21.36969	23.0426	39.83112	41.64159	40.61017
STOX1	2.094697	2.233572	1.961604	0.306627	0.423702	0.29065
STX3	9.400299	8.380538	10.13485	2.260248	3.644467	4.325834
TBX3	0.426014	0.326434	0.357573	4.516156	4.547937	4.750547
TFAP2B	0.57888	0.651586	0.482695	1.971179	1.733262	1.963217
TGFB2	4.16766	3.640564	4.011931	15.23583	16.14925	16.66093
TGFB3	30.8429	28.22994	28.20698	64.43011	65.36925	67.74986
TNFRSF11A	1.668993	1.824798	1.839844	4.890774	5.299934	5.573768
TTK	22.96047	24.21651	23.15277	4.940696	5.176556	5.838467
XBP1	64.97792	64.81932	68.17142	143.667	133.7319	141.4889
BIRC5	60.71144	62.87405	61.45079	15.63261	17.15475	18.74156
BORA	5.916147	5.342473	6.091557	1.29317	0.855645	1.405262
BRSK1	2.165779	2.255654	2.063415	0.612646	0.652981	0.722849
CDK1	160.5851	152.6843	159.891	50.32134	47.60255	52.0064
CDKN2B	2.656072	2.896148	2.005408	31.82536	31.92907	30.61182
CEP135	1.991593	1.934012	1.67138	0.673665	0.676061	0.661896
CEP192	14.10384	13.8097	14.82353	4.318755	4.161137	4.19932
CEP78	14.65682	16.36901	13.59117	5.119322	3.328813	4.54346
CHEK1	23.16898	22.85759	22.70904	4.694255	3.995602	4.222816
CIT	11.74647	12.94598	12.19655	2.644128	2.282684	2.56194
CNTRL	9.395086	9.864534	9.233436	3.916258	4.075438	3.637652
FBXL12	6.048792	6.157124	6.808621	20.0814	19.23509	18.16817
FBXL15	5.023001	4.777298	4.755267	15.74905	16.61239	16.46428
FBXL7	6.98808	7.558629	6.904166	2.775635	2.222664	2.260662
HAUS1	24.73668	23.50116	24.79571	6.179621	6.785613	7.436465
HAUS3	10.47442	10.77826	10.84918	1.909648	2.428387	2.649463
HAUS6	27.22521	28.17567	27.54536	10.08845	10.26297	11.53271
HAUS8	21.06254	21.37935	21.55758	5.646509	6.206003	4.988046
KAT14	22.8195	21.68998	21.66567	8.664042	9.520019	8.893519
KHDRBS1	81.92941	83.39895	82.3934	27.90995	27.32162	29.64991
LCMT1	34.18907	34.87984	36.92661	9.655486	10.64875	10.89395

LOC101751348	0	0.023115	0.024075	0.28103	0.316173	0.317445
MASTL	15.48038	14.71349	14.46909	4.063046	3.715466	4.489508
NEDD1	24.74103	26.49449	26.30484	7.094469	7.903366	7.569943
PLCB1	5.833017	6.069753	6.064602	1.500998	1.407571	1.588427
PLK4	23.04164	22.13776	22.29814	5.494021	5.252618	5.850974
TPD52L1	1.006088	0.964418	1.130019	5.960355	5.403452	5.246897
WEE1	72.73843	74.78642	77.09618	26.85316	28.58078	27.23507
ACVRL1	0.272709	0.462858	0.309906	1.725056	1.982807	1.204948
BMP6	0.438497	0.59244	0.575205	1.566714	1.933435	1.410827
BMPR2	10.88997	10.65465	11.53432	18.43178	18.91335	19.72375
CAV2	2.565392	2.332567	2.452554	7.359858	6.684934	6.430212
CDH13	9.342719	9.35922	9.088796	2.378952	2.269382	2.144823
EGFL7	0.672943	0.473859	0.577796	0.901639	1.057964	1.074429
EGR3	4.442445	5.035618	4.247773	1.075188	1.357423	1.450812
F3	2.641204	1.331265	1.602224	26.64592	30.95588	28.33021
IL10	0	0	0	0.928252	0.602498	0.725906
NRARP	1.908089	2.032732	1.760979	4.494279	5.496868	4.816565
NRP2	21.60672	22.19546	22.0055	40.42377	41.96202	43.26562
POLD4	2.328597	1.631704	2.499196	11.86394	12.37128	11.66058
PRKCA	14.72875	16.05946	14.1996	3.773895	3.422577	3.471134
THBS4	6.500346	6.131888	6.816761	2.003089	1.95603	1.864145
VEGFA	9.993235	8.247996	8.463379	31.44819	29.68081	27.37701
VIP	1.708075	1.958856	1.685373	19.5299	18.87803	19.43382
BTG2	16.33618	16.89127	16.91711	55.72762	57.3818	57.39097
GATA2	0.488275	0.63217	0.46358	16.4174	16.98172	16.17095
ID2	32.69561	33.11159	33.5927	100.386	100.6402	105.0976
KDM2B	24.80432	24.85227	25.13546	7.853876	7.464578	7.769358
LOC107051508	12.95485	11.47082	13.20875	41.70303	42.99675	40.97598
PAX6	1.294749	1.515118	0.997061	0.030548	0.087243	0.023889
SPINT1	12.43587	12.08936	10.97982	57.70936	58.52948	56.58296
BCAT1	32.1348	32.23716	32.2458	12.19437	12.69929	12.88527
CUL4B	73.94275	75.5003	76.49385	28.73578	29.13107	29.69193
DBF4	48.35658	47.06261	47.71524	16.96668	16.02267	18.24605
GPR132	0.013602	0.049059	0.072995	0.298228	0.243345	0.140672
INHBA	1.24909	1.322369	0.900526	10.86084	11.20907	12.20476
LOC101749531	5.522296	6.232803	5.485853	1.674183	1.928816	1.778376
MCM10	7.059995	7.366714	8.083354	1.605467	1.622828	2.068315
NASP	154.1975	154.5584	155.7239	37.66096	35.72299	37.33243
ORC1	7.436958	8.169428	8.677607	3.181197	3.132988	3.347231
ORC5	18.88949	18.91867	18.63276	4.372555	4.397175	5.391229
PIAS1	8.135273	8.446636	8.456021	22.9903	24.09805	24.35693
PPP6C	20.14985	20.3875	22.08005	34.80762	36.35643	33.9701
RCC1	22.69403	21.15205	21.02128	7.497896	7.022114	7.377294
RGCCL	1.53963	1.564048	0.984425	12.1868	11.2709	9.259822

TRIM71	25.19917	26.28107	24.87925	6.748651	6.544039	7.057442
C6orf89	3.008147	3.018142	3.13495	5.412274	5.195266	5.393058
NR4A3	0.143761	0.237926	0.25248	4.211659	4.416348	4.234934
RAD23A	97.37725	94.04773	92.22907	203.3304	201.7153	191.6597
SLC6A4	0.111153	0.076361	0.066276	0.270778	0.428503	0.533302
SERPINB5	0.162308	0.128658	0.241199	3.024126	2.572024	2.405682
SIX5	6.602611	7.503258	6.964923	15.52678	15.29881	16.18132
TACSTD2	0.126467	0.162901	0.101799	1.089296	0.617041	0.413015
INPP5D	0.449149	0.519128	0.325712	1.368759	1.421466	1.704692
RC3H1	8.296216	7.863417	8.448774	14.50536	15.09097	15.35539
TNFRSF21	22.91313	21.257	21.44182	36.45042	37.74324	35.01351
TREMB2	0.244277	0.195783	0.072826	1.771063	2.324827	2.053487
ACVR2B	7.284815	8.303734	6.291334	1.404856	1.57634	1.582681
ALKBH3	16.80117	17.20609	19.31509	6.552439	7.058347	6.011707
ANXA7	3.319248	3.624376	4.118032	22.50927	22.18742	23.93417
BMP4	2.429212	1.916548	2.322022	15.9502	11.9346	12.4991
CD74	8.611417	6.993449	6.821705	17.5103	16.45058	17.1194
CKS2	48.26231	40.91235	45.45179	16.28628	16.1807	17.65581
CLK1	21.26637	23.99025	22.22189	76.79626	74.25754	77.12655
CSPG4	0.412801	0.284789	0.379215	2.187762	2.419933	2.368736
CUZD1	0.167659	0.131066	0.167532	0	0	0
DLX5	0.045104	0.348592	0.314657	2.660608	3.814491	2.995128
DTYMK	54.14739	54.0588	54.62749	12.22663	10.32179	13.07752
E2F8	21.70964	21.90012	23.0294	6.872107	9.034162	7.989252
ECD	15.58025	14.88723	13.49039	48.83482	51.47243	50.96917
ENPEP	0.022558	0.046491	0.032281	1.161861	1.068005	1.096858
ENTPD5	5.234631	4.804886	3.981478	12.82853	13.32251	14.04651
FAM83A	0.076807	0.266225	0.157374	0.05103	0.037853	0.007601
FAM83B	0.795213	0.839296	0.800315	1.80697	1.894452	1.961981
FAM83D	16.75959	15.62253	15.75916	3.357471	3.544232	3.529637
FGF5	0.799689	0.895101	0.76209	0.035987	0.201814	0.097561
GLI2	7.780238	7.298374	7.710611	1.551724	1.875458	1.617959
GRHL2	1.070604	1.2225	1.149039	3.455198	3.407855	3.823174
HLCS	18.63694	18.69693	18.33883	4.680119	4.11294	4.534249
HOXB4	0.497072	0.363946	0.126353	9.218374	9.729356	9.597618
IRF2	2.173358	2.036732	2.264972	47.1981	49.65589	46.69252
LIPML5	0.020124	0.145159	0.086392	2.542188	1.745507	2.672607
LOC107049174	0.085267	0.052719	0.009151	0.151337	0.332818	0.241336
LOC107049543	3.358172	2.88043	3.25003	6.627037	7.945076	6.846517
LOC107055388	0.740694	0.660739	0.93734	1.385033	1.186652	1.480257
LOC107055390	4.297791	4.045329	3.973674	47.72581	49.70479	45.3477
LOC121108911	25.39143	24.57826	24.96303	7.091195	9.034058	9.070401
LRP2	46.68188	49.0447	47.87015	5.96139	6.503743	7.296691
LY6CLEL	0.873967	0.096493	0.301499	1.727141	0.676848	0.71355

LY6E	84.50684	86.56499	83.39428	28.15727	30.78373	30.0271
MELK	26.74502	29.16727	26.78208	5.737198	4.438866	4.629156
MXD1	4.480615	4.435806	4.87971	15.64154	15.48851	15.53485
MYH10	62.64092	63.17107	63.2362	23.41082	24.07049	24.18813
NAA60	2.151752	2.394716	2.052795	5.122026	5.018561	5.122035
NCF1C	2.414189	2.596718	1.815632	6.071269	6.419693	6.119406
OGFOD1	26.1133	26.1578	26.47584	10.48801	9.340071	10.18606
PDK1	124.4613	126.2888	122.9313	21.02082	21.54303	21.52875
PDXK	5.274484	5.069559	4.423124	16.02345	16.60548	15.97431
PICALM	8.322519	8.088124	8.488102	20.53196	21.33754	21.49846
PIM1	19.23159	19.70933	17.31096	59.85104	61.15804	58.65656
PLCE1	9.01827	9.010985	9.459226	1.272618	1.308716	1.570557
POLA1	28.70212	29.18582	30.37449	7.793142	7.803353	8.501332
POLR3G	1.096596	1.760724	1.505383	2.689147	3.414834	2.845575
PRMT5	89.51483	86.89248	87.0758	31.37427	30.89982	28.54535
RHBDF1	1.613363	1.880394	1.951029	3.833788	4.231312	4.216264
SBDS	23.39996	22.81432	22.05235	42.49558	43.49635	44.17122
SDCBP2	0.226487	0.042434	0.220982	2.665549	2.902136	5.087962
SH2D2A	0.008745	0.018024	0.028158	0.209998	0.331868	0.218962
TCF7L2	3.142775	3.37888	2.707648	1.219681	1.346298	1.213227
TOP2A	60.231	59.3502	59.55434	10.90321	11.07119	11.53903
TRIM27.1	0.038492	0	0.041312	15.02983	15.54598	15.25235
TRIM27.2	0.111096	0.034344	0.011923	0.579938	0.325226	0.302346
TRIM39.2	0	0	0.016478	0.112206	0.016647	0.150423
TSPAN1	12.27393	13.11727	11.35777	32.57938	35.22113	34.04084
USP13	19.31526	19.70047	20.4125	5.840179	5.553699	6.996916
USP28	22.37489	24.09093	22.49732	6.088025	6.837525	6.645246
WNT2B	0.301	0.265863	0.092301	0.448935	0.963525	0.436891
XRCC5	31.61956	31.09073	32.62707	9.367169	9.82705	11.74198
ARX	2.591685	3.022549	2.74397	0.868418	1.044257	1.048458
BCL6	5.727857	7.421125	6.472395	18.50581	18.25101	16.96289
CDCA7	14.78943	14.21728	14.43595	4.579181	4.713797	4.716007
CGTL	0.489485	0.390665	0.391434	1.177395	1.347608	0.99257
DBH	0.260908	0.158872	0.127283	10.62349	11.43119	10.3798
FA2H	1.870797	1.253078	1.706677	11.52374	10.20955	10.6749
FES	0.228988	0.277004	0.213708	1.101807	0.863573	0.812857
FZD5	11.43788	10.76452	11.61357	2.139814	1.983264	1.927267
IER5	17.82782	15.14926	17.51166	49.26679	49.21075	46.23051
JUP	39.2247	40.40171	39.4031	108.608	110.4646	105.2857
KLF4	4.985007	4.019226	4.585904	22.06487	18.99447	18.93969
MITF	3.875138	3.998723	3.696289	9.369753	9.747869	9.68278
NDRG1	29.33077	29.26938	28.7204	60.68591	58.07998	58.84423
NKX2-3	2.641204	1.913078	2.711457	3.856514	4.378542	3.979459
NUAK1	15.01661	17.42547	15.98855	33.92504	32.8999	33.28585

PLCD1	4.080804	3.907213	4.233391	8.634918	8.600681	9.437043
PTGS2	4.04118	3.775329	3.75939	12.27619	12.39328	12.01871
S100A11	65.36272	65.10124	68.71703	434.6237	422.7752	397.8925
SAT1	96.58457	100.3277	97.86225	1214.852	1214.086	1206.582
SGK2	0	0.007519	0	0.091417	0.134494	0.071489
SIX3	10.32072	10.71122	13.50319	0.359173	0.506219	0.374504
SLA	0.314521	0.409398	0.322335	0.723404	0.746132	0.821215
STAT6	5.473339	4.937019	5.09476	9.544352	11.2324	10.19935
TCF7	3.935007	4.210891	5.393943	1.923374	1.805049	1.647555
TCFL5	24.36572	25.38139	24.96436	5.604725	5.106319	5.052678
TMIGD1	0.065056	0	0	0.101881	0.08817	0.300985
TNC	10.21309	10.57918	10.75305	60.44114	60.41677	61.26972
TNK2	5.69451	5.669878	4.747394	12.38515	12.06868	11.53
IFT88	20.8699	22.36828	22.89177	7.383719	8.331945	7.443373
PCNT	15.01969	15.81749	15.70093	4.886574	4.736002	5.328976
ZNF503	1.412573	1.132152	1.389724	8.182955	8.381062	7.037231
CFLAR	1.449958	1.358931	1.72753	4.758329	4.5266	5.13285
LOC121109701	0	0	0	0	0.119594	0.240149
TNFAIP3	0.563496	0.699859	0.630243	9.1931	9.219109	8.842944
BG8	0.114419	0.235812	0.28069	7.133319	7.354834	7.33104
BTN3A3L2	0	0	0	0.60658	0.669309	0.968473
LOC121106920	0.010475	0	0.022485	0.065617	0.045429	0.136836
MOGL	0.071591	0	0	0.772348	0.905584	0.896238
BLM	16.25712	15.9332	16.63778	6.111115	6.20154	6.481076
CCR2	0.059342	0.148508	0.263856	2.823377	2.665543	2.417868
RIPK3	0.891663	1.124784	0.923989	2.134712	2.583621	2.158889
SYK	0.336612	0.163975	0.151078	0.600634	0.457868	0.40641
AKR1E2	0.153672	0.305399	0.424106	3.575489	3.617961	4.504797
CCL5	0	0.547727	0.684562	7.991038	6.224062	6.133376
IL6	0.750326	0.239956	0.360995	1.053492	1.59901	1.013963
LOC425137	0.148262	0.289479	0.209374	4.472644	5.110205	4.502159
HEY2	2.141546	1.982591	2.39677	3.790181	4.619054	4.051698
MEF2C	0.607686	0.734588	0.522601	1.21602	1.16148	1.340015
TBX20	2.954022	2.128547	2.979738	0.463775	1.035511	1.039677
TBX5	0	0.025029	0.032585	0.564213	0.717611	0.449485
ZFPM2	1.072258	1.197529	0.983657	0.400259	0.584538	0.313008
C1QL4	14.3311	11.50682	12.76092	6.560696	3.235106	4.724538
FTH1	159.3488	152.6305	156.2573	1675.259	1630.269	1627.579
IL8L1	0.561986	0.723891	0.201053	17.11302	15.5378	16.64543
IL8L2	2.757643	5.120155	5.146101	67.10059	64.45877	60.87773
NLRC3	0.337647	0.581894	0.393623	0.893442	0.94047	0.659076
PEX2	13.56579	13.65315	13.91741	3.091034	3.539559	2.991057
SOD2	59.86395	56.97724	59.34297	104.3071	99.08043	100.5622
TP53INP1	25.82603	26.12762	25.45298	10.84152	10.5918	10.01686

GPC3	0.999174	0.600615	0.983011	3.412042	3.611139	3.33108
IFT172	11.17316	10.0578	10.73584	3.952193	4.075147	4.766616
IFT74	42.13734	39.83259	40.10932	14.30545	16.33448	15.88886
IFT80	33.62561	34.68601	34.17894	11.40143	12.79594	12.05739
LOC101747554	0	0	0.009779	0.209289	0.069156	0.109111
LOC420903	0.111096	0.091585	0.131158	1.565832	0.999767	1.366605
MTSS1	9.389508	9.306811	9.911606	16.27797	16.92521	17.87106
RUNX3	0.577423	0.528081	0.31761	1.635231	1.463424	1.382881
SOX2	9.879148	7.501222	6.988891	0.904752	1.1812	1.024231
SOX9	5.707052	6.655281	6.125164	17.83772	20.61953	17.9759
