# Supplementary material for: Comparison of primordial germ cell differences at different developmental time points in chickens
Source: Anim Biosci. 2024 Aug 5;37(11):1873–86. doi: 10.5713/ab.24.0283 (PMC11541041; doi:10.5713/ab.24.0283)
Supplement: Supplementary file 10 [file ab-24-0283-Supplementary-Table-10.pdf]

Table S10. qRT-PCR primers sequence of related genes

Gene	F (5'-3')	R (5'-3')
ACTB	ACCGCAAATGCTTCTAAAC	GACTGCTGCTGACACCTTC
CVH	AGCACAGGTGGTGAACGAA	TGCTGGTGGATGGTAGGTT
POU5F3	AAACGCAGGACCAGCATCG	AGCAGCCGCTTGCCTTTCT
