# Supplementary material for: Comparison of primordial germ cell differences at different developmental time points in chickens
Source: Anim Biosci. 2024 Aug 5;37(11):1873–86. doi: 10.5713/ab.24.0283 (PMC11541041; doi:10.5713/ab.24.0283)
Supplement: Supplementary file 11 [file ab-24-0283-Supplementary-Fig-1.pdf]

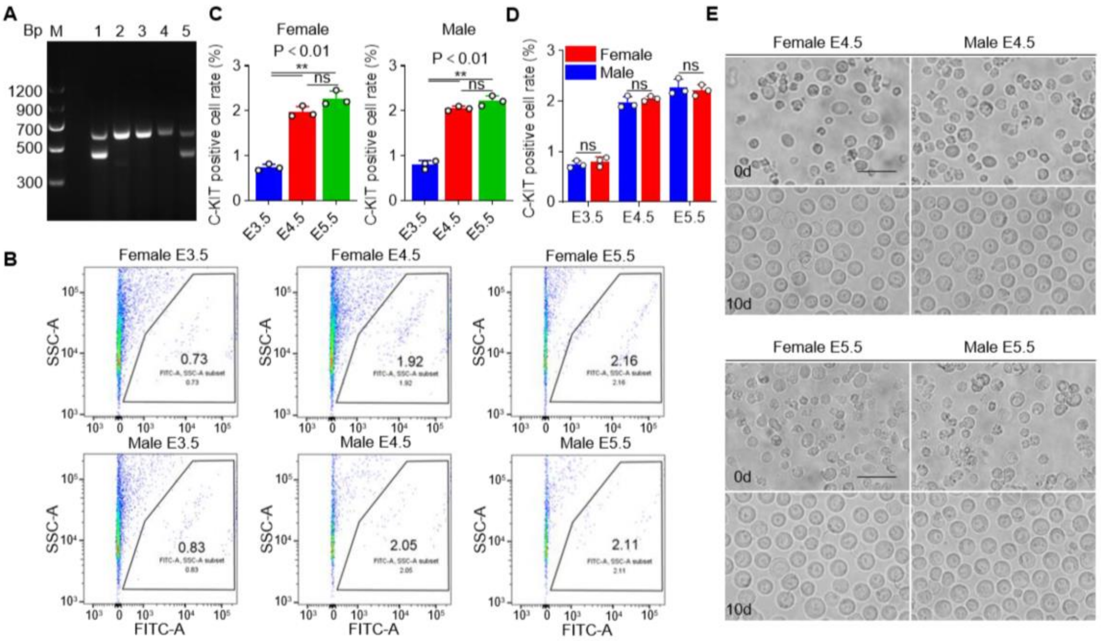

Figure S1. Isolation and identification of chicken PGCs. A. Sex identification of PGCs by amplifying *CHD1*. B~D. The proportion of PGCs in the genital ridges at E3.5-E4.5 was analyzed by flow cytometry. E. Morphological observation of female and male PGCs at E4.5 and E5.5 after isolation and purification. Scale bar: 60 μm.
