# Supplementary material for: Comparison of primordial germ cell differences at different developmental time points in chickens
Source: Anim Biosci. 2024 Aug 5;37(11):1873–86. doi: 10.5713/ab.24.0283 (PMC11541041; doi:10.5713/ab.24.0283)
Supplement: Supplementary file 12 [file ab-24-0283-Supplementary-Fig-2.pdf]

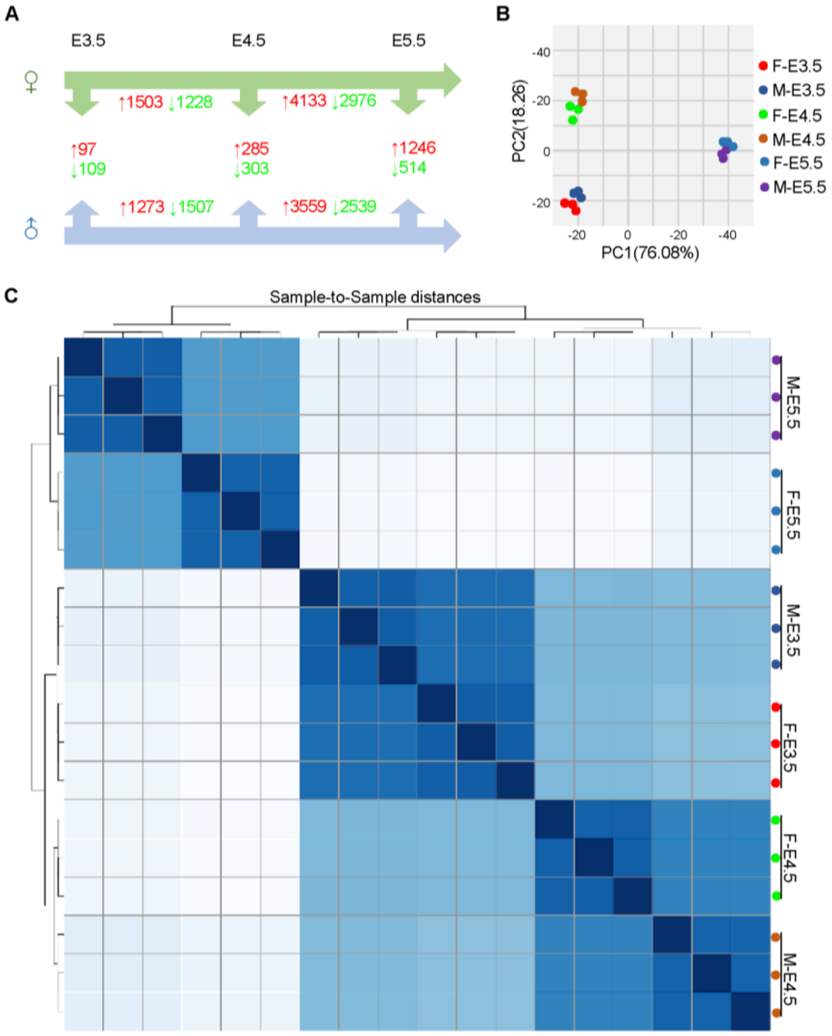

Figure S2. Large differences were found among PGCs of E3.5、4.5 and 5.5, but not between males and females. A. Statistics of DEGs during during the development of female and male PGCs from E3.5 to E5.5. Red represents up-regulated expression and green represents down-regulated expression. B~C. PCA and Pearson's correlation analysis of the differences among female and male PGCs at E3.5, E4.5 and E5.5.
