# Supplementary material for: Comparison of primordial germ cell differences at different developmental time points in chickens
Source: Anim Biosci. 2024 Aug 5;37(11):1873–86. doi: 10.5713/ab.24.0283 (PMC11541041; doi:10.5713/ab.24.0283)
Supplement: Supplementary file 13 [file ab-24-0283-Supplementary-Fig-3.pdf]

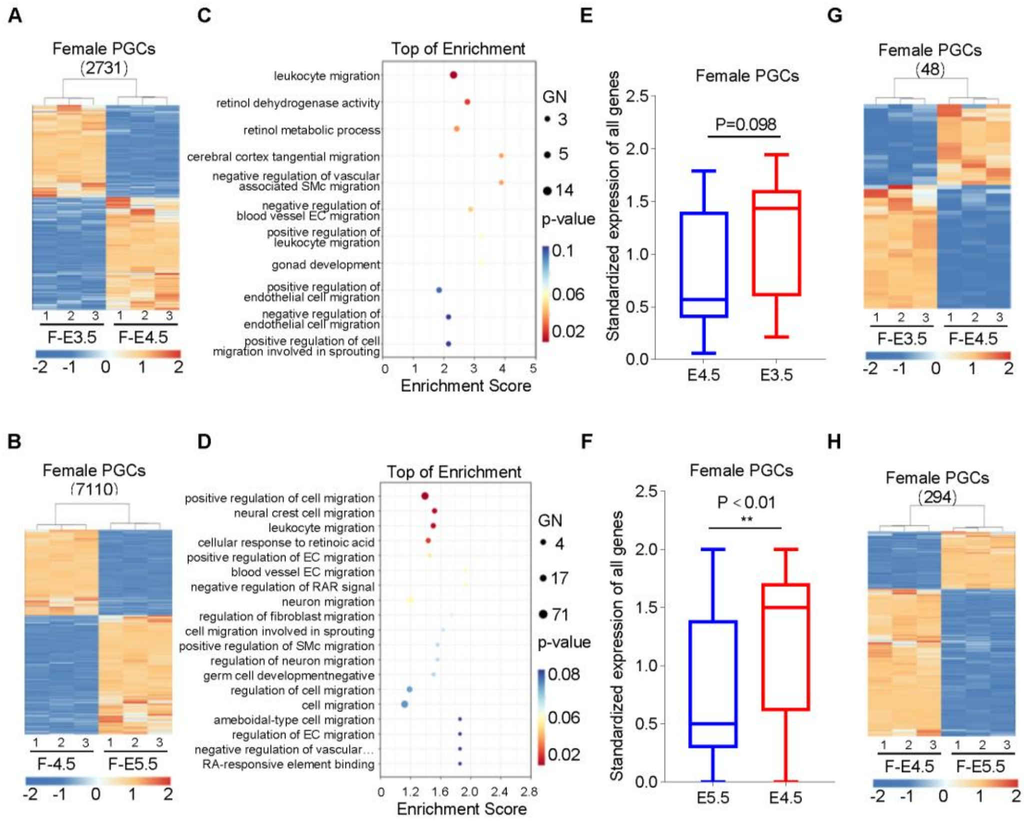

Figure S3. Evaluation of the migration ability of female PGCs during development. A, B. Heat map analysis of DEGs during the development of female PGCs from E3.5 to E4.5 (A) and from E4.5 to E5.5 (B). C, D. GO analysis of DEGs during the development of female PGCs from E3.5 to E4.5 (C) and from E4.5 to E5.5 (D). E, F. Expression analysis of genes related to germline transmission ability (including genes related to migration and gametogenesis) during the development of female PGCs from E3.5 to E4.5 (E) and from E4.5 to E5.5 (F). G, H. Heat map analysis of genes related to germline transmission ability during the development of female PGCs from E3.5 to E4.5 (G) and from E4.5 to E5.5 (H).
