# Supplementary material for: Comparison of primordial germ cell differences at different developmental time points in chickens
Source: Anim Biosci. 2024 Aug 5;37(11):1873–86. doi: 10.5713/ab.24.0283 (PMC11541041; doi:10.5713/ab.24.0283)
Supplement: Supplementary file 14 [file ab-24-0283-Supplementary-Fig-4.pdf]

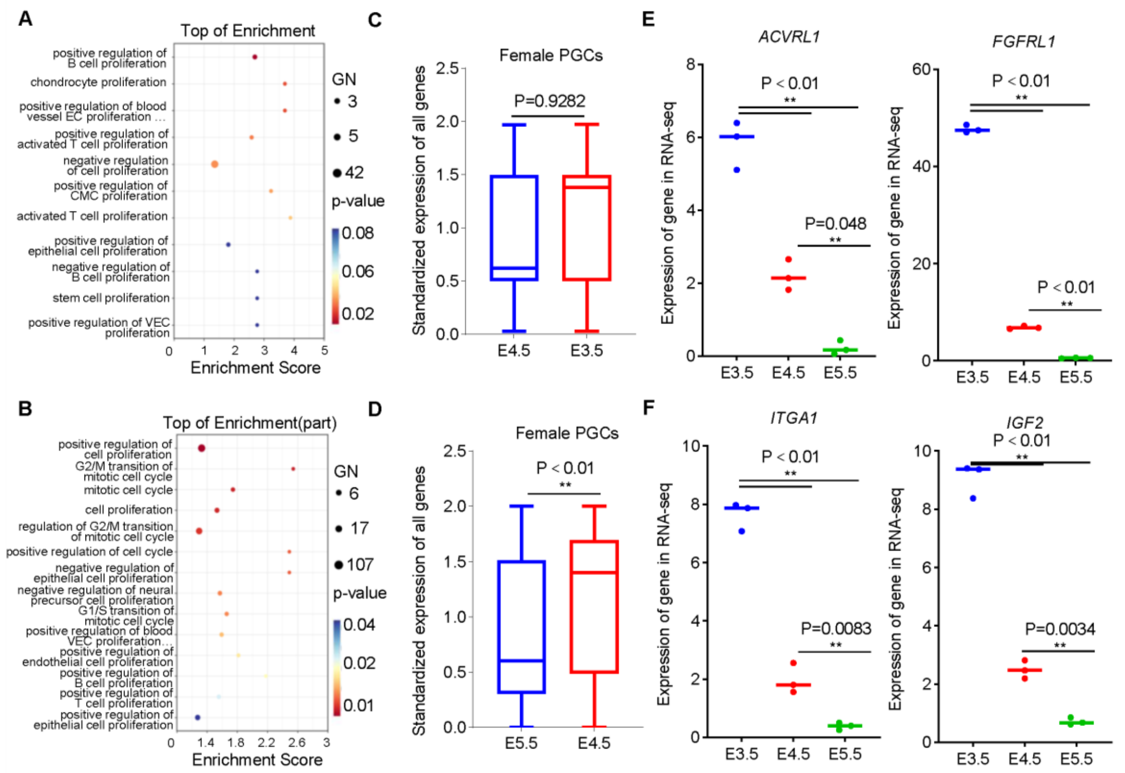

Figure S4. Changes in the proliferation ability of female PGCs during development. A, B. GO analysis of DEGs during the development of female PGCs to screen terms related to cell proliferation. C, D. Expression analysis of genes related to proliferation during the development of female PGCs from E3.5 to E4.5 (C) and from E4.5 to E5.5 (D). E, F. Specific expression analysis of genes related to cell proliferation during the development of female PGCs from E3.5 to E4.5 (E) and from E4.5 to E5.5 (F).
