# Supplementary material for: Comparison of primordial germ cell differences at different developmental time points in chickens
Source: Anim Biosci. 2024 Aug 5;37(11):1873–86. doi: 10.5713/ab.24.0283 (PMC11541041; doi:10.5713/ab.24.0283)
Supplement: Supplementary file 15 [file ab-24-0283-Supplementary-Fig-5.pdf]

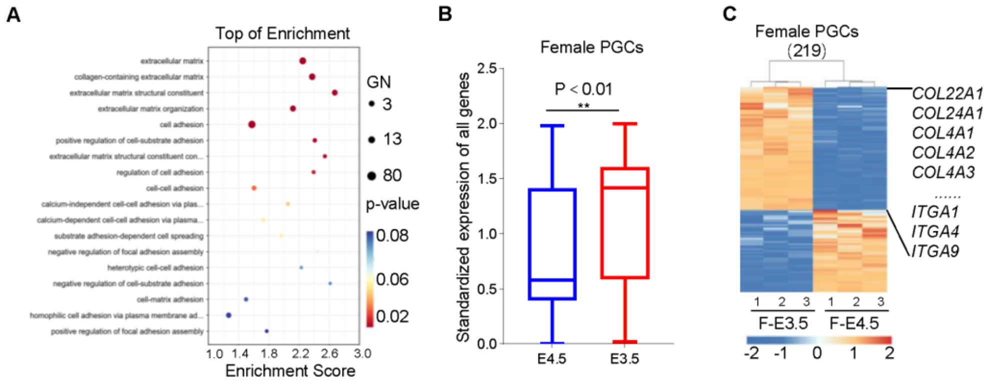

Figure S5. Changes in cell adhesion and intercellular communication during the development of female PGCs. A, B. GO analysis of DEGs during female PGC formation to screen terms related to cell adhesion and intercellular communication. C, D. Expression analysis of genes related to cell adhesion and intercellular communication during the development of female PGCs from E3.5 to E4.5. E, F. Specific expression analysis of genes related to cell adhesion and intercellular communication during the development of female PGCs from E3.5 to E4.5.
