# Supplementary material for: Comparison of primordial germ cell differences at different developmental time points in chickens
Source: Anim Biosci. 2024 Aug 5;37(11):1873–86. doi: 10.5713/ab.24.0283 (PMC11541041; doi:10.5713/ab.24.0283)
Supplement: Supplementary file 16 [file ab-24-0283-Supplementary-Fig-6.pdf]

A

	3.5d		4.5d		5.5d	
	Number of GO Term	Number of Gene	Number of GO Term	Number of Gene	Number of GO Term	Number of Gene
Related to cell proliferation	9	8	5	16	9	73
Related to cell adhesion	5	15	1	2	19	152
Related to germ line transmission	2	4	3	6	7	50

B

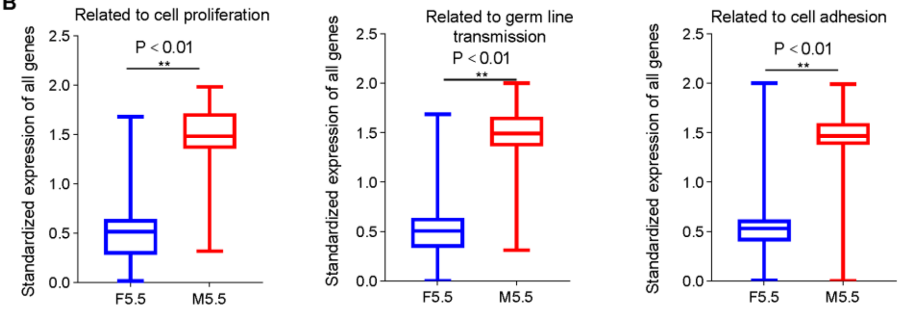

Figure S6. Comparison of differences between female and male PGCs at different developmental time points. A. Quantitative statistics of the DEGs and GO terms related to cell proliferation, germline transmission ability and cell adhesion of female and male PGCs at E3.5, E4.5 and E5.5. B. Analysis of differences in cell proliferation, germline transmission ability and cell adhesion between female and male PGCs at E5.5.
