# Supplementary material for: Comparison of primordial germ cell differences at different developmental time points in chickens
Source: Anim Biosci. 2024 Aug 5;37(11):1873–86. doi: 10.5713/ab.24.0283 (PMC11541041; doi:10.5713/ab.24.0283)
Supplement: Supplementary file 18 [file ab-24-0283-Supplementary-Fig-8.pdf]

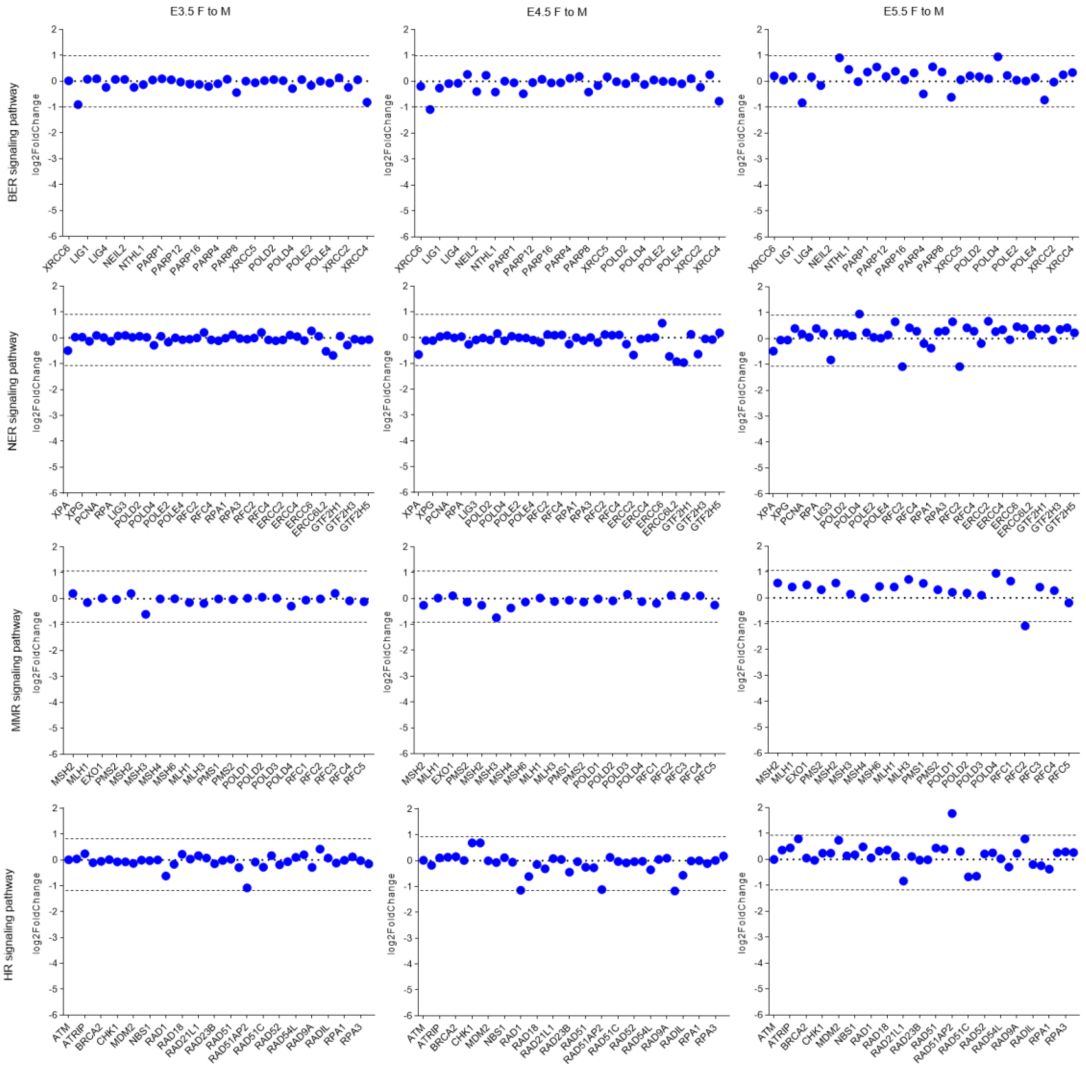

Figure S8. Analysis of signaling pathways related to damage repair among female and male PGCs at different developmental time points.
